# 3D-PP: A Tool for Discovering Conserved Three-Dimensional Protein Patterns

**DOI:** 10.3390/ijms20133174

**Published:** 2019-06-28

**Authors:** Alejandro Valdés-Jiménez, Josep-L. Larriba-Pey, Gabriel Núñez-Vivanco, Miguel Reyes-Parada

**Affiliations:** 1 Center for Bioinformatics, Simulations and Modelling, Universidad de Talca, 3460000 Talca, Chile; 2 PhD Program on Computer Architecture, Universitat Politécnica de Catalunya, 08034 Barcelona, Spain; 3 DAMA-UPC, Universitat Politécnica de Catalunya BarcelonaTech, 08034 Barcelona, Spain; 4 Facultad de Ciencias de la Salud, Universidad Autonóma de Chile, 3467987 Talca, Chile; 5 School of Medicine, Faculty of Medical Sciences, Universidad de Santiago de Chile, 9170022 Santiago, Chile

**Keywords:** conserved patterns, similarity, 3D-patterns

## Abstract

Discovering conserved three-dimensional (3D) patterns among protein structures may provide valuable insights into protein classification, functional annotations or the rational design of multi-target drugs. Thus, several computational tools have been developed to discover and compare protein 3D-patterns. However, most of them only consider previously known 3D-patterns such as orthosteric binding sites or structural motifs. This fact makes necessary the development of new methods for the identification of all possible 3D-patterns that exist in protein structures (allosteric sites, enzyme-cofactor interaction motifs, among others). In this work, we present 3D-PP, a new free access web server for the discovery and recognition all similar 3D amino acid patterns among a set of proteins structures (independent of their sequence similarity). This new tool does not require any previous structural knowledge about ligands, and all data are organized in a high-performance graph database. The input can be a text file with the PDB access codes or a zip file of PDB coordinates regardless of the origin of the structural data: X-ray crystallographic experiments or in silico homology modeling. The results are presented as lists of sequence patterns that can be further analyzed within the web page. We tested the accuracy and suitability of 3D-PP using two sets of proteins coming from the Protein Data Bank: (a) Zinc finger containing and (b) Serotonin target proteins. We also evaluated its usefulness for the discovering of new 3D-patterns, using a set of protein structures coming from *in silico* homology modeling methodologies, all of which are overexpressed in different types of cancer. Results indicate that 3D-PP is a reliable, flexible and friendly-user tool to identify conserved structural motifs, which could be relevant to improve the knowledge about protein function or classification. The web server can be freely utilized at https://appsbio.utalca.cl/3d-pp/.

## 1. Introduction

Most drugs interact with more than one molecular target [1,2]. This fact is usually considered an undesired feature since it might be related to the side effects of pharmacological treatments. However, current trends in drug discovery have put hope and considerable effort into the development of multitarget compounds, due to the improved efficacy and safety profiles shown by some promiscuous drugs [3,4,5,6,7,8]. In this context, several computational approaches to predict the polypharmacological profile of either novel or known drugs have been developed, most of which are based on two main methodological strategies. In the first case, methods are based on ligand characteristics, for example, the search of compounds showing similar pharmacological/molecular activities with known drugs, those that represent the ligands as a bi-dimensional graph and look for similarities in databases using graph-based techniques and those based on the three-dimensional (3D) similarities of ligands. The second approach is centered on target(s) features and involves methods that use the known 3D structure of proteins to perform inverted docking, structure-based pharmacophore searching and the evaluation of binding sites similarities [8,9,10].

The usefulness of assessing structural similarities of ligand binding sites in different proteins, aimed to target clustering or drug development, is supported by the fact that the structure of proteins is several times more conserved than their sequences [11,12,13]. Furthermore, even in those cases where a close evolutionary relationship exists between two proteins, it might be possible that their global sequences and structures were not conserved and only share partial 3D-patterns, which define, in most cases, the function of such proteins. Indeed, the comparative analysis of important 3D protein patterns such as binding sites, catalytic sites and protein-protein interaction motifs, have been recently used to, for example, identify putative off-targets of known drugs, the design of polypharmacological compounds and drug re-purposing [14,15]. For these aims, several computational tools have been developed [16,17,18,19,20,21,22,23,24,25], which, in general, require a known query (ligands/binding sites) for their searching processes. Thus, these algorithms usually utilize (only) the orthosteric binding site in proteins, annotated motifs and/or previously known functional residues to make similarity assessments. This represents a weakness of the current tools, since some evidence indicates that a conserved 3D arrangement of amino acids might be enough to consider such a 3D-pattern as functionally relevant, even if no prior knowledge of their biological activity is available (e.g., protein cavities/pockets that may serve as allosteric sites) [20,26,27,28,29,30,31].

Thus, unveiling and comparing all local structural patterns (including those unknown or previously unobserved) into a set of protein structures could be more informative for the discovery, search and characterization of conserved 3D-patterns than exploring only previously known sites. In a recent report [32], we described a strategy for the exhaustive searching of similar 3D-patterns between two protein structures, which allowed the discovery of some conserved structural residue arrangements between proteins that differ in their function, structure and tissue localization but that share the same endogenous ligand and perform complementary physiological functions [33]. This type of finding, along with the increasing availability of structural data (more than 130,000 protein structures in the Protein Data Bank [34] and more than 3 million homology models in the SWISS-MODEL Repository [35]), represent an opportunity to use and develop structure-based methods for the classification, description and discovery of conserved 3D amino acid patterns among multiple protein structures.

Here we present 3D-PP, a new free access web server designed to discover all conserved 3D amino acid patterns among a set of protein structures. The pre-processing modules of 3D-PP were developed in Python language and all data generated are processed and organized automatically in a scalable, high-performance graph database [36]. Remarkably, this kind of database has shown better performance than relational databases, particularly when problems must be realistically modeled through, for example, the use of properties in a graph mode analysis [37,38,39,40,41].

## 2. Results

To demonstrate the applicability of 3D-PP, in the following sections we show the results of two different examples in which the existence of known and unknown 3D-patterns are assessed in a set of proteins. Also, as a benchmark analysis, we tried to replicate the same experiments with other available tools.

### 2.1. Known Small 3D-Patterns

**Figure 1:**
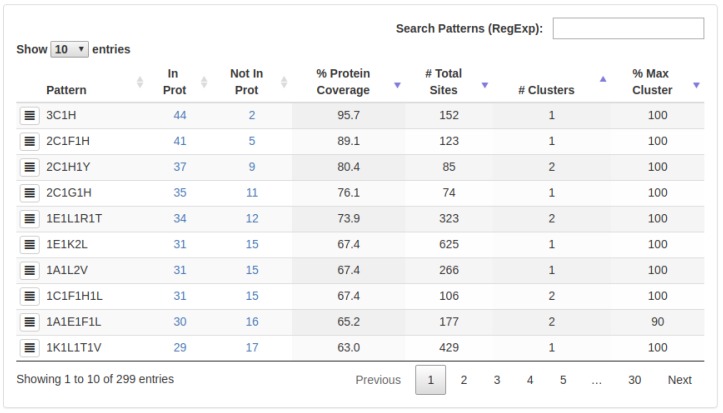
Coverage of 3D-patterns identified in the Zinc finger C3H1-type protein structures. Figure 1 shows the list of all 3D-patterns detected and several criteria for filtering.

We used a dataset of 46 protein structures, all of which contain the PROSITE Zinc finger C3H1-type motif (https://prosite.expasy.org/PDOC50103) (PDBids: *1m9o, 1rgo, 2cqe, 2d9m, 2d9n, 2e5s, 2fc6, 2rhk, 2rpp, 3d2n, 3d2q, 3d2s, 3jb9, 3tp2, 3u1l, 3u1m, 3u9g, 4c3b, 4c3d, 4c3e, 4cyk, 4ii1, 4yh8, 5elh, 5elk, 5gmk, 5lj3, 5lj5, 5lqw, 5mps, 5mq0, 5mqf, 5u6h, 5u6l, 5u9b, 5wsg, 5y88, 5ylz, 5z58, 6bk8, 6dnh, 6eoj, 6exn, 6fbs, 6ff4, 6fuw*; range of PDB resolutions: 1.5 to 5.9 Å). This sequence motif is composed of three cysteines and one histidine amino acids, which are located in the primary sequence as defined by the following regular expression *C-x(8)-C-x(5)-C-x(3)-H*. At a structural level, this motif represents a small 3D-pattern, is highly conserved and shows chemical coordination of the residues with one Zinc ion. Usually known as Zinc finger, this pattern is essential for the folding stabilization of this kind of protein structure [42]. After the simultaneous evaluation of these 46 protein structures, 3D-PP identified 737,793 sites corresponding to 43,305 3D-patterns organized in 47,203 clusters. As shown in Figure 1, the 3C1H was the most represented 3D-pattern with a protein coverage value of PCv = 95.7%.

**Figure 2:**
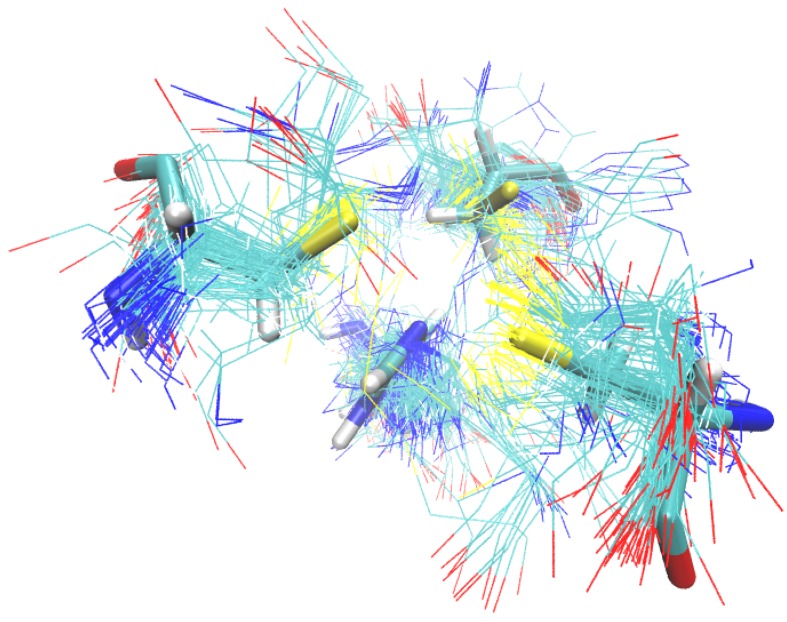
Structural alignment of the 152 sites detected in the cluster 3C1H-1. This result is delivered by 3D-PP using an interactive Jsmol Viewer.

**Figure 3:**
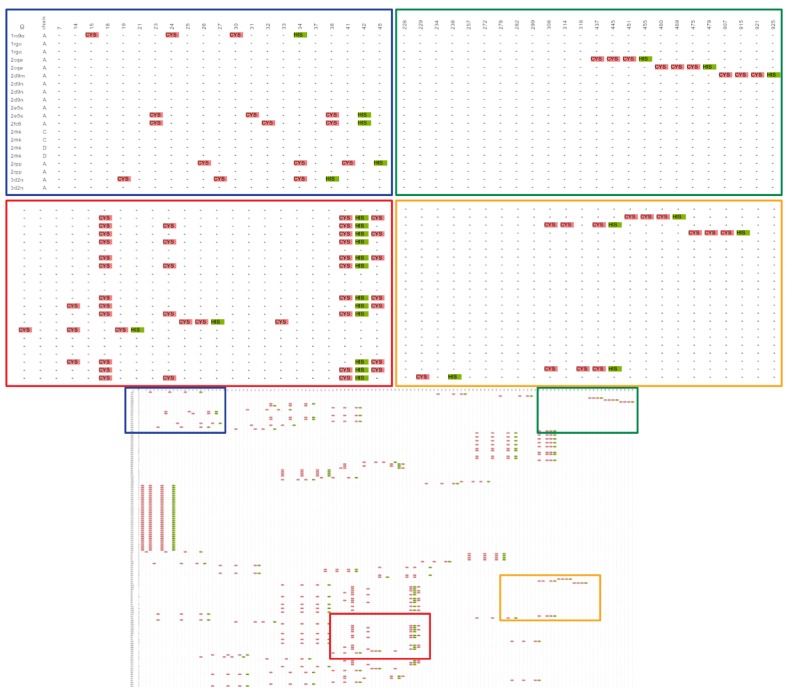
Sequence representation of the structural alignment of the 152 sites detected in the cluster 3C1H-1. This result is delivered by 3D-PP. The diffuse lower box shows the entire representation of the sequence alignment of the 152 sites found. The blue and green boxes show a zoom denoting the PDBids, the chain and the original PDB residue number of each site. The red and orange boxes exemplifies that some 3D-patterns found exhibit the expected sequence order (C-x(8)-C-x(5)-C-x(3)-H), whereas other sites, while having the same structural orientation, do not match with the canonical Zinc finger C3H1-type motif.

This value means that this 3D-pattern was found in the vast majority of the proteins’ structures (44 of 46 proteins). Also, this pattern grouped in only one cluster (cluster coverage CCv = 100%; Figure 1), which denotes that in those 44 proteins structures, there is at least one site whose 3D topological conformation does not exceed the root mean square deviation (RMSD) threshold defined by the user (4.5 Å in this example; Supplementary Data, Figure S1). This RMSD threshold is an important input parameter of our software because it allows to discriminate between 3D-patterns that contain similar components (i.e., amino acid residues) but exhibit different topological conformations (i.e., they are not in the same spatial localization/order). Thus, in 3D-PP even though several 3D-patterns might show a high level of protein coverage (PCv), they will appear grouped in different clusters with low coverage (CCv) if they show a high structural and/or topological diversity. In this example, only one cluster formed by 152 sites was detected in the 3D-Pattern 3C1H, denoting high structural conservation (Figure 2) and irregular sequence localization. As shown in Figure 3, the common 3D-pattern can appear in different locations of the sequences (blue and green boxes in Figure 3). Also, even though the 3D-pattern found corresponds to sites structurally conserved it can occur with differential sequence order in the global protein sequence (red and orange boxes in Figure 3).

Interestingly, 122 of the 152 detected sites were confirmed by the presence of the *C-x(8)-C-x(5)- C-x(3)-H* pattern in the primary sequence of the proteins analyzed and also by the appearance of the respective Zinc ion in coordination with three cysteine and one histidine amino acid in the corresponding crystal structures (confirmed using the PDBsum server [43]). The remaining sites detected by 3D-PP have similar structural features to the confirmed sites but either the protein structure does not have a co-crystallized Zinc ion or the sequence localization of the residues in the sites does not match with the corresponding PROSITE pattern (Table 1 and Supplementary Data, Table S1).

In the detailed analysis of the new sites unveiled by our software, we remark the following particular cases:

#### 2.1.1. Putative New Zinc Ion Coordination Sites

**Figure 4:**
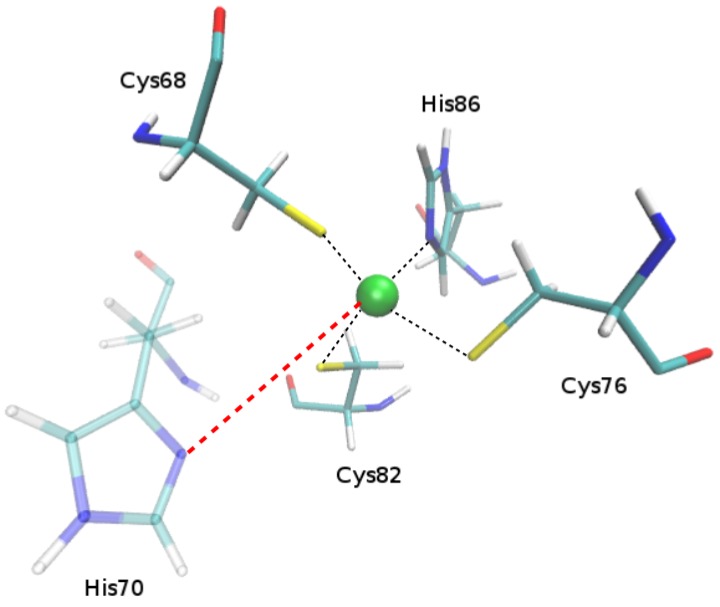
Putative new site discovered by 3D-PP. In the figure the residues that form the new site detected are shown. The green sphere represents the Zinc ion.

For the protein structure with PDBid:2D9N, three sites were detected by 3D-PP. Two of them were confirmed at both sequence and structural levels and the third was only found by our software (Supplementary Data, Table S1, PDBid:2D9N). This new site, which is formed by the residues Cys68, Cys76, Cys82 and His70, shares the 3 cysteine residues with a known/confirmed site but involves a different histidine residue (His70 instead His86). As shown in Figure 4, this new identified site might keep the coordination of the Zinc ion in cases in which, for example, a punctual specific mutation of the residue His86 occurs. It should be noted that the calculated pKa of His86 (which forms the canonical Zinc coordination site) and His70 (the new putative site) was below 6, indicating that both residues are mostly deprotonated and therefore are able to establish coordination with the Zinc ion. Thus, in theory, the Zinc ion might be “moving” between both sites, since both offer a favorable environment to stabilize its binding. In the same line, the other 29 sites with similar features were discovered by our software (Supplementary Data, Table S1, Tag “New Site” in column “Comments”).

#### 2.1.2. Promiscuous Binding Sites

Another remarkable result was the identification of two Cadmium ion binding sites that appeared in the same 3D-pattern cluster as the Zinc ion binding sites. These two sites belong to the crystal structure of the Essential Transcription Antiterminator M2-1 Protein of the human respiratory syncytial virus (PDBid:4c3d). As shown in the Supplementary Data, Figure S2, this structure effectively contains two Cadmium ions co-crystallized, which are coordinated with 3 cysteine and 1 histidine residues. These results are in agreement with previous reports that show that Zinc ions can be interchanged by Cadmium ions in some enzymes [44], indicating that this 3D-pattern can act as a promiscuous binding site. It is worth pointing out that 3D-PP does not use the information about ligand/ions co-crystallized with the protein structures and only works with the 3D-patterns found from the virtual grid of coordinates (see Materials and Methods Section).

#### 2.1.3. Not Found Patterns

As we indicated above, in 2 of the 46 protein structures submitted it was not possible to identify 3D-patterns with the components 3C1H. These proteins, namely pre-mRNA-processing-splicing factor 8 of Human (PDBid:5MQF) and Yeast (PDBid:5LQW), were the biggest structures evaluated in this set of data. Both structures are biological assemblies obtained through cryogenic electron microscopy at a resolution of 5.9 Å and 5.8 Å , respectively. As we confirmed in our detailed analysis, low resolution—in general—limits the possibility of obtaining all the coordinates of residue side chains, some hydrogen bonds and small ligands such as metal ions. In the case of these proteins, most chains have only the atomic coordinates for the backbone and unfortunately our software cannot detect 3D-patterns without considering the side chain of protein residues.

### 2.2. Serotonin Target Proteins

**Figure 5:**
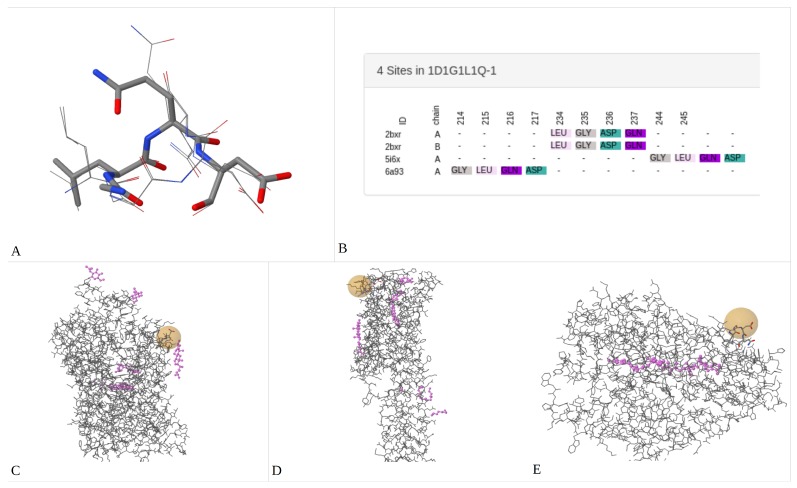
Conserved 3D-pattern among the serotonin target proteins. **A** shows the structural alignment of the four sites forming the 3D-pattern 1D1G1L1Q. **B** shows a representation of a sequence alignment of the structurally aligned sites that form the 3D-pattern 1D1G1L1Q. **C**, **D** and **E** show the structural localization of the sites forming the 3D-pattern 1D1G1L1Q on the global structure of SERT, 5HTR2A and MAO-A, respectively.

Serotonin (5-Hydroxytryptamine; 5-HT) is a biogenic amine which is found in the gastrointestinal tract, blood platelets and the central nervous system (CNS). In the CNS, 5-HT acts as neurotransmitter and is released into the synaptic cleft where it interacts with specific 5-HT receptors (5-HTRs) to activate different signal transduction pathways [45]. After that, 5-HT is pumped back into the nerve terminals by the 5-HT transporter (SERT) and/or is metabolized by the enzyme monoamine oxidase type-A (MAO-A) [46]. Even though these three types of proteins (5-HTRs, SERT and MAO-A) have distinct functions, different sequences and diverse structural folding, they share 5-HT as the primary endogenous ligand. As observed in the matrix of amino acids’ sequence identity (Supplementary Data Table S2), the range of pair-wise alignment among these proteins does not exceed 22%. In addition, their multiple sequence alignment (MSA; Supplementary Data Figure S4) only shows 15 residues conserved but with very disperse localization. Therefore, biologically relevant results cannot be obtained with these sequence alignment methods. To test our software, we submitted the crystal structures of the human SERT (PDBid:5I6X), MAO-A (PDBid:2BXR) and 5-HT${}_{2A}$ receptor (PDBid:6A93) using the following input parameters: St: 2 Å, Rt: 5 Å, RMSDt: 4.5 Å, Dt: 2 Å and Mc: 80%. Despite protein differences, 3D-PP was able to detect several 3D-patterns with a 100% of coverage; one of them, the 3D-pattern 1D1G1L1Q, shows two clusters with 100% and 33% of CCv (Cluster Coverage), respectively. The first has four sites composed of one aspartate, one glycine, one leucine and one glutamine amino acids. These sites have an RMSD lower than 2.5 Å, show a similar 3D topological conformation (Figure 5A), their residues are unsorted on each primary sequence (Figure 5B) and their structural localization corresponds, for SERT and 5-HTR2A, at the extracellular side (Figure 5C,D), whereas in MAO-A, the site was detected in the protein surface (Figure 5E). The presence of aspartate residues on these sites could be significant because this type of amino acid has been shown to be critical, for example, in the inner binding site (Asp-98 [47]) and the antidepressant binding site of SERT (Asp-400 [48]), in the binding sites of the 5-HT receptors (Asp-155 [49]) and in the substrate/inhibitor cavity of MAO-A (Asp-328, Asp-132 [50]). Thus, these sites could represent a useful starting point for the design of allosteric multi-target drugs ([51]).

### 2.3. Finding/Discovering Unknown 3D-Patterns on Homology Model Structures

**Figure 6:**
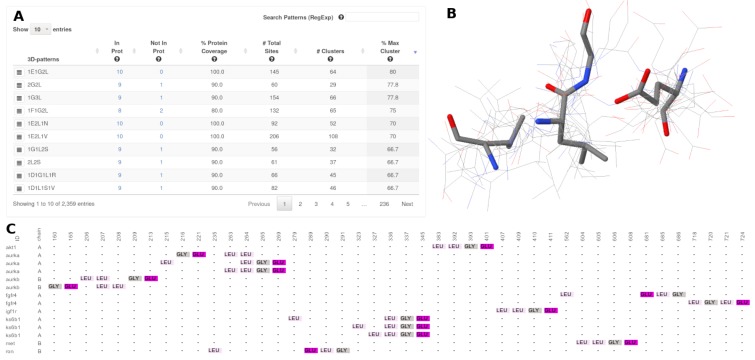
Coverage of 3D-patterns detected in the homology models of over expressed proteins in some cancer types. **A** shows the list of conserved 3D-patterns. **B** shows the structural alignment of the sites forming the 3D-pattern 1E1G2L. **C** shows a representation of a sequence alignment of the structurally aligned sites that form the 3D-pattern 1E1G2L.

In this case, we tried to discover conserved 3D-patterns among 10 protein structures generated through the SwissModel server (homology models). All of these proteins are over-expressed in different types of cancer (breast, prostate, lung, gastric, etc) and correspond, for example, to the insulin-like growth factor 1 receptor, the macrophage-stimulating protein receptor and the aurora kinase B, among others [52]. After the assessment with 3D-PP, our results showed the existence of several common 3D-patterns in these proteins (high PCv coverage; Max PCv = 80%) but many of them showed high structural or topological diversity (low CCv coverage). Nevertheless, the most structurally conserved 3D-pattern has a cluster with sites occurring in 8 of the 10 homology models submitted (cluster 1E1G2L-14, CCv = 100%; Figure 6A). The conservation of this 3D-pattern (1E1G2L; one glutamate, one glycin and two leucine amino acids; Figure 6B), is attractive since it might represent an event of convergent evolution which could be useful for establishing a functional annotation [53], the design of new poly-pharmacological anticancer drugs [4] and/or protein structure-based diagnosis [54]. As discussed above, this conserved 3D-pattern was detected in spite of their non-conserved sequence order (Figure 6C).

### 2.4. Comparison with other Methods for the Search and Description of Amino Acid Patterns

Table 2 summarizes some features of computational tools aimed at the search of structural protein patterns, with comments regarding the results obtained when the same data set used in this work was evaluated.

In general terms, none of the software indicated in Table 2 was able to perform the same analysis as 3D-PP. Nevertheless, they were included in the benchmark, since they are the currently available algorithms with most similar performances/objectives as compared with 3D-PP. In spite of this, it seems probable that with using MMDB and VAST+ tools in combination with ProBIS (and a series of additional processing), results similar to those of 3D-PP may be obtained.

## 3. Materials and Methods

3D-PP discovers conserved 3D protein patterns among an arbitrary set of structures uploaded by the user. For this, the user must define the following five threshold parameters:

- Spacing Threshold (St): This value is used to create the Virtual Grid of Coordinates and defines, how broad and rigorous will be the exploration of 3D-patterns. For instance, a St = 0.5, means that every 0.5 Å in the 3D space of each protein structure, a new virtual coordinate of reference will be created. In all cases analysed in this work (Zinc finger C3H1-type containing proteins, serotonin target proteins and structures obtained from homology models), St values were 0.8, 2 and 0.8 Å, respectively.
- Radius Threshold (Rt): This term represents the limits of the size of the 3D-patterns searched. Low Rt values are used to detect small binding sites ( e.g., 3 Å), whereas high values allow identification of bigger sites (e.g., 7 Å). In the two cases analyzed in this work (Zinc finger C3H1-type containing proteins, serotonin target proteins and structures obtained from homology models), Rt values were 3, 5 and 2 Å, respectively.
- Displacement Threshold (Dt): This value is used to expand the size and shape for the exploration of the 3D-patterns. By default, this value is set in 0, which means that only the spherical 3D-patterns are searched. If the user changes this value; for example, Dt = 2, two new virtual centers will be considered for the searching of 3D-patterns. This option allows the obtaining of seven new elliptical/oval zones that will be explored to detect non spherical 3D-patterns (Supplementary Data, Figure S3).
- RMSD Threshold (RMSDt): This value is used for clustering the 3D-patterns detected and represents a measure of structural variability for the sites composing each 3D-pattern. As mentioned in the Results, this parameter allows the comparison of a 3D-pattern with those, containing the same components (i.e., amino acid residues), previously found by 3D-PP. Thus, if the new site exceeds the threshold values defined by the user (RMSDt) when comparing it with the previously found site, a new cluster of the same 3D-pattern is created. Otherwise, the new 3D-pattern is included in the same cluster as that previously found. Therefore, this parameter is crucial for 3D-PP accuracy since it allows discrimination between 3D-patterns that contain similar components but exhibit a different topological conformations (i.e., amino acid residues which are not in the same spatial localization/order).
- Minimum Coverage (Mc): This value allows the showing of only the 3D-patterns with a coverage value equal to or higher than Mc.

The sites, each one defined as a structural arrangement of residues, form different structural clusters in the same 3D-pattern. Each cluster has a central feature named coverage (Cv), which represents the conservation level among the evaluated proteins. For example, a Cv value of 100% denotes a cluster formed by sites occurring in all the assessed proteins and whose structural orientation/conformation show high similarity. Detailed information about the architecture and the essential components of 3D-PP are shown in the Supplementary Data, Figure S5.

### 3.1. Grid of Virtual Coordinates

**Figure 7:**
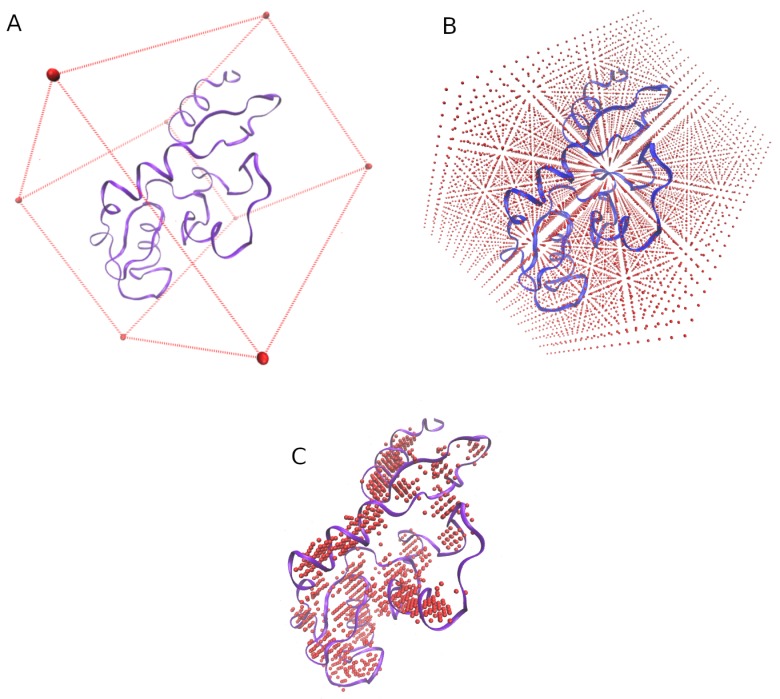
Grid of Virtual Coordinates (GvC). Letters **A**, **B**, and **C**, show the process of creating a Grid of Virtual Coordinates for each protein structure. The red spheres represent the reference coordinates from which the searching of 3D-patterns will be done.

**Figure 8:**
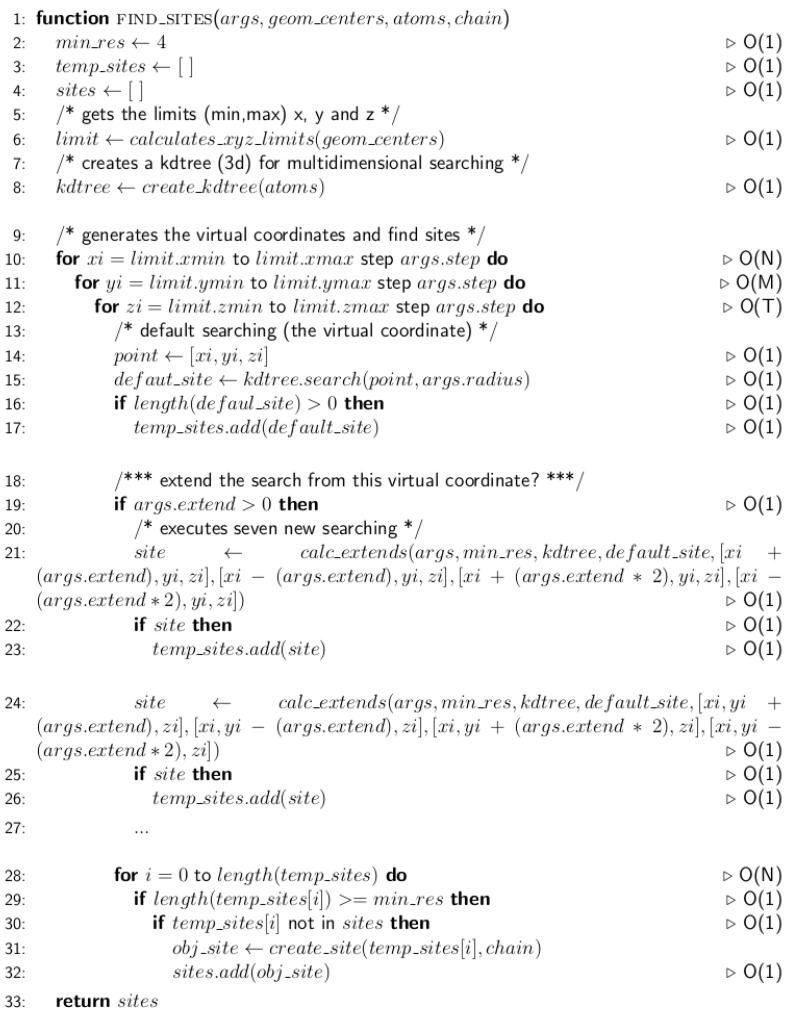
Pseudocode of the *FIND_SITES* function of 3D-PP. Figure 8 shows the pseudocode of the function for the searching of 3D-patterns without any previous knowledge about the ligands or binding sites in the protein structures. On each line of the algorithm is indicated the computational complexity.

One grid of virtual coordinates (GvC) is modeled for each protein structure submitted ( Figure 7). This GvC, generated by the function FIND_SITE (Figure 8), is used for the searching of the 3D-patterns and confers to 3D-PP the ability to prescind from any previous knowledge about the ligands or binding sites in the protein structures.

Briefly, each GvC is constructed as follows:

- the min and max values of the X, Y, and Z axis of each structure are obtained.
- a virtual box whose size is determined by the previous values is defined (Figure 7A).
- the virtual box is filled by reference coordinates (*x*, *y*, *z*) distanced between them by an user-defined value (e.g., St = 2 Å, Figure 7B).
- only the reference coordinates that show at least four residues surrounding (at a user-defined distance, e.g., Rt = 5 Å) will be considered for the final grid (Figure 7C.)

### 3.2. Protein Preprocessing

This step represents the core of 3D-PP since it is responsible for the identification of all possible sites (arrangements of structurally related amino acids) and generates all input data for the graph database. It is worth noting that the identification of the sites is independent of the order of the amino acids sequences of proteins. This pre-processing considers all chains of each protein separately and utilizes the GvC previously generated. The GvC is used as follows:

- residues of the proteins surrounding each coordinate of the GvC until a user-defined distance (Rt) are grouped.
- groups of residues with at least four components are considered as a site.
- a vector with the list of residues is defined for each site. Then, the sites are transformed into a representation of components through a sorted alphabetical list which contains the one letter code of the amino acid and the amount of occurrences of the same amino acid (e.g., the site “H31:I32:K10:K90:L11:L12:L7:P92:S3:S8:T9” is transformed into “1H1I2K3L1P2S1T”).
- If two different sites match in their representation of components (e.g., “1H1I2K3L1P2S1T”), the RMSD between these two sites is measured. If the RMSD exceeds the threshold values defined by the user (RMSDt), a new cluster of the same 3D-pattern is created. On the contrary, a new site is added to the current cluster. This step is implemented to avoid two sites having the same components but different 3D conformations, being grouped in the same cluster. It should be noted that if the user set too permissive RMSDt values (high values), there are more possibilities for grouping sites with different structural topologies; thus, many false positives can occur.

### 3.3. Creation of the Graph Databases

For each protein structure submitted, a new graph database is created simultaneously using parallel programming approaches. In these databases, the new sites identified are stored as a new node (SITE node; Supplementary Data, Figure S6A). Then, the main graph database, which is an extension of the first model, is used for the unification of data (e.g., 3D-patterns and sites of the protein 1, 3D-patterns and sites of the protein 2, etc.). For this, all the SITE node attributes are used to create or connect the corresponding PATTERN nodes, CLUSTER nodes and finally, to establish the edges SITE_IN_CLUSTER, CLUSTER_IN_PATTERN, and PATTERN_IN_PROTEIN (PATTERN_IN_PROTEIN; Supplementary Data, Figure S6B).

It is important to note that the PATTERN node with the highest amount of PATTERN_IN_PROTEIN edges represents the 3D-pattern with the highest coverage value. Moreover, if this PATTERN node has few CLUSTER_IN_PATTERN edges it is possible to estimate that the sites that are part of this 3D-pattern have a high level of structural and topological conservation. On the contrary, many CLUSTER_IN_PATTERN edges indicate a high level of structural diversity.

### 3.4. Result Visualization

The first level of results shows, as a graph and dynamic data tables, all 3D-patterns discovered in the set of protein structures submitted. Additionally, the user can search sub-patterns of interest through a simple regular expression query. For instance, the regular expression ⌃2C.*2H$, will detect all the sub-patterns that begin with 2C and finish with 2H, with any character in between, which represents a 3D-pattern containing precisely two cysteines, two histidines and any other amino acids.

Once the measures have been done, every 3D-pattern has the following ranking features available:

- InProt: The number of proteins in which a specific 3D-pattern was detected.
- NotIn: The number of proteins in which a specific 3D-pattern was not detected.
- %ProteinCoverage(PCv): Level of conservation of a 3D-pattern in the set of proteins evaluated. The PCv is calculated as follow:
  PCv=InProt/(amountofproteinssubmitted)
  A high PCv value (e.g., 80%) indicates that a pattern containing a certain type of residues is found in many proteins (e.g., 80% of the proteins analyzed). It is worth noting that the sites composing a 3D pattern found do not necessarily exhibit the same structural topology in all the proteins in which such a pattern occurs.
- #TotalSites: Amount of sites (arrangement of residues) which are part of a specific 3D-pattern.
- #Clusters: This value represents the structural variability of a 3D-pattern. Thus, a low number of clusters denotes low variability and, on the contrary, a high number of clusters is indicative of several structural conformations (with different topologies) of sites forming a 3D-pattern.
- %Max.Cluster: Represents the cluster with the highest coverage on each 3D-pattern.

The second level of results appears in the exploration of a particular 3D-pattern. Here, all clusters identified for the selected 3D-pattern are shown as a dynamic data table, where the following features are available:

- #Sites: Amount of sites (arrangement of residues) which are part of a specific cluster.
- InProt: The number of proteins that contain a particular 3D-pattern.
- %ClusterCoverage(CCv): Level of conservation of a cluster in the set of proteins belonging to a particular 3D-pattern. The CCv is calculated as follows:
  CCv=InProt/(amountofproteinsintoaparticularcluster)
  A high *CCv* value (e.g., 80%) indicates that a pattern with the same structural topology is present in most of the proteins (80%) of the corresponding cluster.
- SequenceAlignment: This button shows a multiple sequences-based alignment of the residues of each site of a specific cluster.
- StructuralAlignment: This button displays a jsmol viewer with multiple structural-based alignments of the residues of each site of a particular cluster.

The last level of results is displayed selecting a particular cluster. Here, all the sites grouped into a specific cluster are shown as a dynamic data table and the following features are available:

- Site: Information of the name and number of the residues forming the site.
- Protein: Name of the protein where the site was detected. This variable can be the PDBid or the name of the file, in the case of homology models.
- Chain: The chain where the site was detected.
- RMSD: Root mean square deviation of atomic positions of the particular site against the reference site.
- SiteID: Referential coordinate of the GvC from where a specific site was detected.
- ViewSite: This button shows a jsmol viewer loading the protein and highlighting the residues corresponding to a particular site.

## 4. Conclusions

In this work, we present 3D-PP, a new free access web server for discovering and recognition of all similar 3D amino acid patterns among a set of protein structures. Our software has three main features that confer competitive advantages as compared with other similar computational tools: **(a)** 3D-PP does not require previous structural knowledge about ligand(s), motif(s) or binding site(s); **(b)** 3D-PP utilizes a scalable, high-performance graph database; **(c)** 3D-PP can be used with protein structures from both experimental biophysics techniques (X-ray crystallography, NMR, etc.) and in silico homology modeling. Also, the results are shown as simple and intuitively dynamic lists of sequence/structural patterns that can be further analyzed within the web page.

We performed three representative types of uses of 3D-PP. **(I)** In the first case, using a set of protein structures containing the small 3D-pattern knows as Zinc finger, our software was able to detect almost all (98%) Zinc finger C3H1-type contained in the PROSITE database and described in crystal structures. Also, 3D-PP unveiled several new sites that have similar structural features to the known sites but which neither have a Zinc ion in the original structure nor a match between the sequence of these sites and the established sequence pattern for this type of motif. Thus, our results indicate that 3D-PP discovered new putative Zinc ion binding sites. As discussed in the Results, some of these new identified sites might serve to enhance the robustness of a crucial biological structure-derived function, by keeping the coordination of the Zinc ion in cases in which, for example, a punctual specific mutation might occur; **(II)** In the second case, we discovered some conserved 3D-patterns in the serotonin target proteins. This finding is significative considering that these proteins (5-HTRs, SERT and MAO-A) have distinct functions, different sequences and diverse structural folding; **(III)** In the third case, we found some conserved 3D-patterns in a set of protein structures coming from the in silico homology models methodologies. Considering that the X-ray structures solved until March 2019 reach a coverage of nearly 50% of the human proteome, the use of homology models substantially improves the scope of these kinds of structure-based methods. In this case for example, our criteria of selection was as ample as “Proteins overexpressed in different types of cancer’,’ which indicates the versatility of 3D-PP.

It is important to mention at least two limitations of 3D-PP. First, it should be noted that to identify two (or more) 3D-patterns as conserved, 3D-PP considers only sites that contain the same components (amino acid residues). It is known that, for instance, some promiscuous drugs/ligands can interact with more than one target even if the corresponding binding sites are not composed of identical amino acids but of residues with similar properties (e.g., hydrophobicity, acid or basic character, aromatic character, etc.). Therefore, 3D-patterns with “similar” structural and functional properties, but with a different composition, will not be detected by 3D-PP. The other limitation is that 3D-PP gives no information about the accessibility/drugability of the conserved 3D-patterns identified. Therefore, if a 3D-pattern is either embedded into the protein structure or in a relatively inaccessible location, it could be unproductive to try to develop compounds aimed to act at that site. Beyond these limitations, and considering as a basic idea that protein structure is more conserved than sequence, 3D-PP appears to be a flexible and user-friendly tool for identifying conserved structural motifs, which could be relevant to improve our knowledge of protein function or classification.

## Figures and Tables

**Table 1:** Number of sites containing the Zinc finger C3H1-type motif at the sequence (PROSITE) and structural (3D-PP and PDBsum) levels. The last column (A & B & C) shows those sites that satisfy the sequence pattern C-x(8)-C-x(5)-C-x(3)-H (**A**), those discovered by our software that matched with the previously described sites (**B**) and those in which PDBsum shows coordination with the Zinc ion (**C**).

Item	PROSITE (A)	3D-PP (B)	PDBsum(C)	A & B	A & C	B & C	A & B & C
**amount of sites**	125	152	124	123	124	122	122
**%**	100%	–	–	94.8%	99.2%	97.6%	97.6%

**Table 2:** Comparative table of similar tools.

Tool	X-ray/Homology Model	Require Known Pattern/Ligand	Comments
Catalytic site identification—a web server to identify catalytic site structural matches throughout PDB [20]	Yes/No	Yes	The most similar section to replicate our experiments with this tool is “Find CSA catalytic sites in your proteins.” However, it was not possible to obtain the results because only one PDB structure can be processed at the same time. When we tried to upload a homology model the server returned a “Server Error (500)”.
MMDB and VAST+ [55]	Yes/No	Yes	This tool allows the finding of 3D-patterns of amino acids in only one PDB structure at the same time. It is not possible to identify conserved 3D-patterns on a set of protein structures.
IMAAAGINE [19]	Yes/No	Yes	This tool allows searching 3D-patterns of amino acids in the entire PDB database. The user must define a structural description of query. It is not possible to identify conserved 3D-patterns on a set of protein structures.
PatternQuery [23]	Yes/No	Yes	This tool allows for the searching of 3D-patterns of amino acids in the PDB database or in a particular data set of protein structures. The user must define a detailed structural description of the query (known 3D-pattern). It is not possible to identify conserved 3D-patterns on a set of protein structures.
ProBiS [24]	Yes/Yes	No	This tool search all the 3D-patterns of amino acids associated to a ligand or functional annotations, present in a queried protein structure. Then these 3D-patterns are searched on the entire PDB database. It is not possible to identify conserved 3D-patterns on a set of protein structures.
Geomfinder [32]	Yes/Yes	No	This tool compares all the possible 3D-patterns of amino acids of one protein structure with all the possible 3D-patterns of a second protein structure. The identification of the 3D-patterns works separately on each pair of protein structures, and the results are not matched. Therefore, it is not possible to identify conserved 3D-patterns in a set of protein structures.
MultiBind [30]	Yes/Yes	No	This tool identifies similar 3D-patterns among a list of PDBids. With this tool, we could find conserved 3D-patterns, but the server did not work with our data sets. In the case of the homology models, the measures can not be assessed because our structures do not contains ligands. The server returns the following comment “No valid ligand. Can not define the query binding site”.

## Supplementary Materials

Supplementary materials can be found at https://www.mdpi.com/1422-0067/20/13/3174/s1.
