# Supplementary material for: 3D-PP: A Tool for Discovering Conserved Three-Dimensional Protein Patterns
Source: Int J Mol Sci. 2019 Jun 28;20(13):3174. doi: 10.3390/ijms20133174 (PMC6651053; doi:10.3390/ijms20133174)
Supplement: Supplementary file 1 [file ijms-20-03174-s001.pdf]

Supplementary Data

Figure S1: RMSD values of the sites occurring in the cluster 3C1H-1.

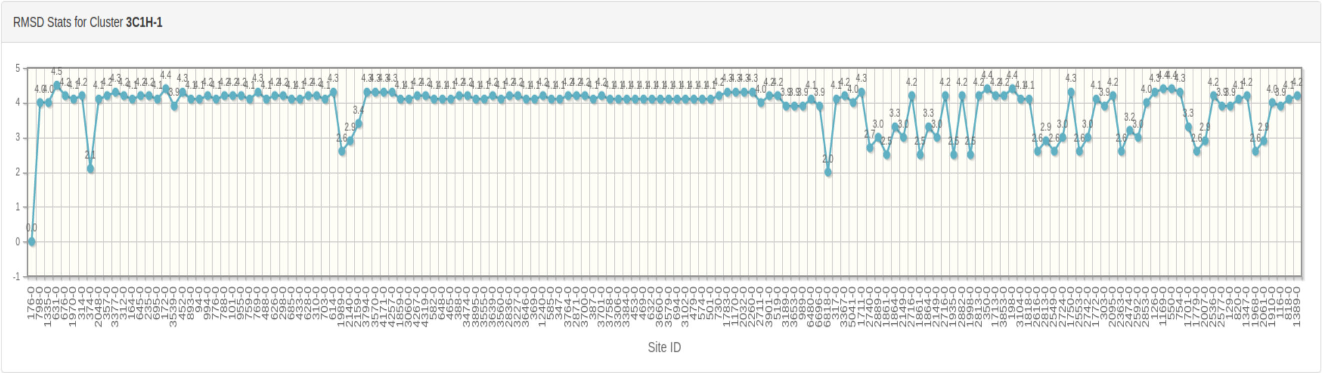

Figure S1: The Y-axis the RMSD values are shown and in the X-axis the ID of the sites that are part of the cluster 3C1H-1 are listed (full results https://appsbio.utalca.cl/3d-pp/results/1556639834.1218/).

Figure S2: Sites detected that correspond to the Cadmium ion binding site of the chain A and B of the PDBid:4c3d.

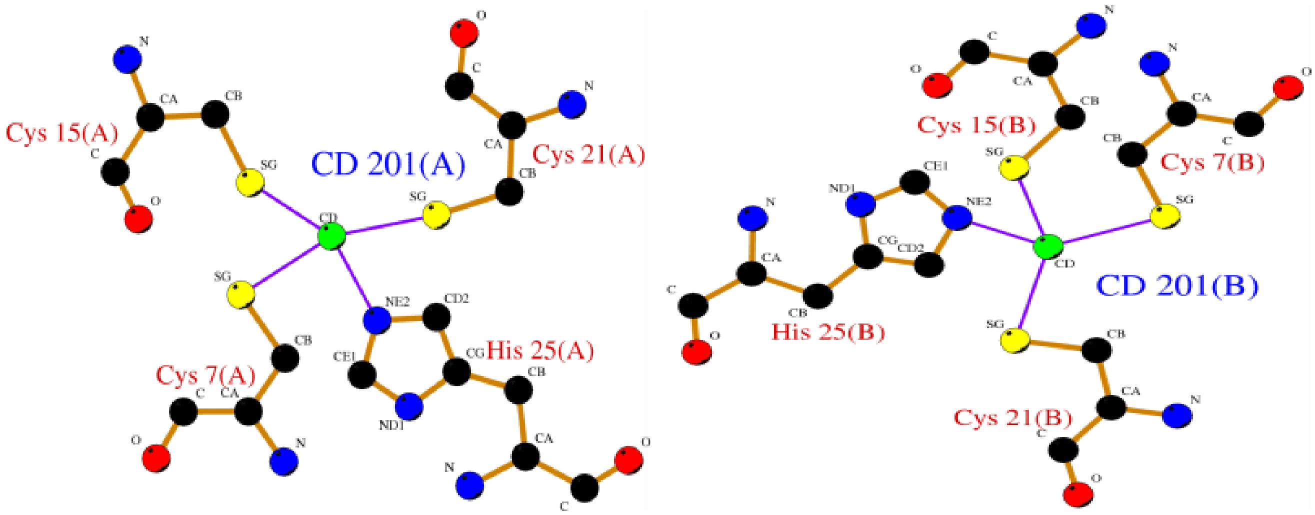

Figure S2: Two green spheres represent the Cadmium ions which are being coordinated by the residues present at the new sites discovered by 3D-PP (images obtained from PDBsum).

Figure S3: Representation of the expanded searching method implemented on 3D-PP to find non-spherical 3D-patterns.

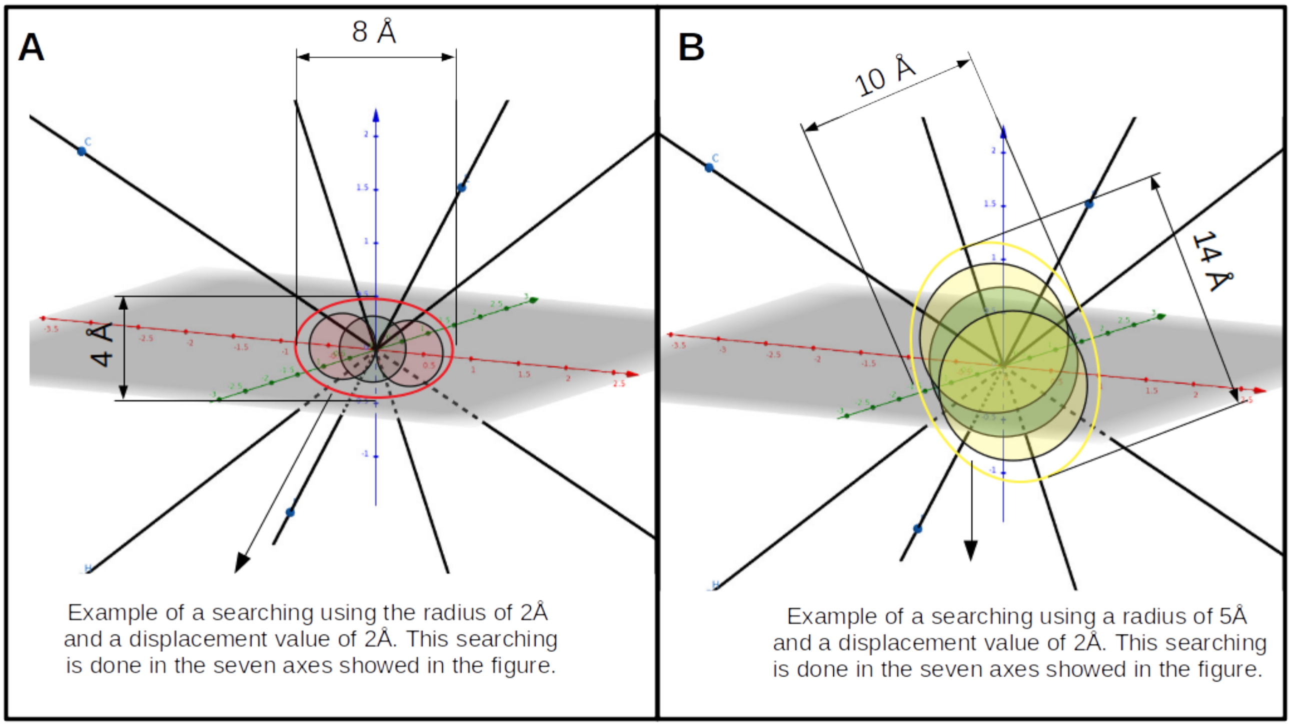

Figure S3: For both cases, A and B, the translucent green sphere represents the center of the searching space for each dummy atom of the Virtual Grid of Coordinates. By default, the search only considers spherical 3D-patterns on that space, but if the user changes the "Displacement threshold" parameter, seven different searching spaces are used for the analysis. In the Figure two examples of "Displacement threshold" values are depicted. Example A shows an elliptical shape of searching, whereas example B shows an oval shape of searching.

Figure S4: Multiple sequence alignment among SERT, MAO-A and 5-HTR2a

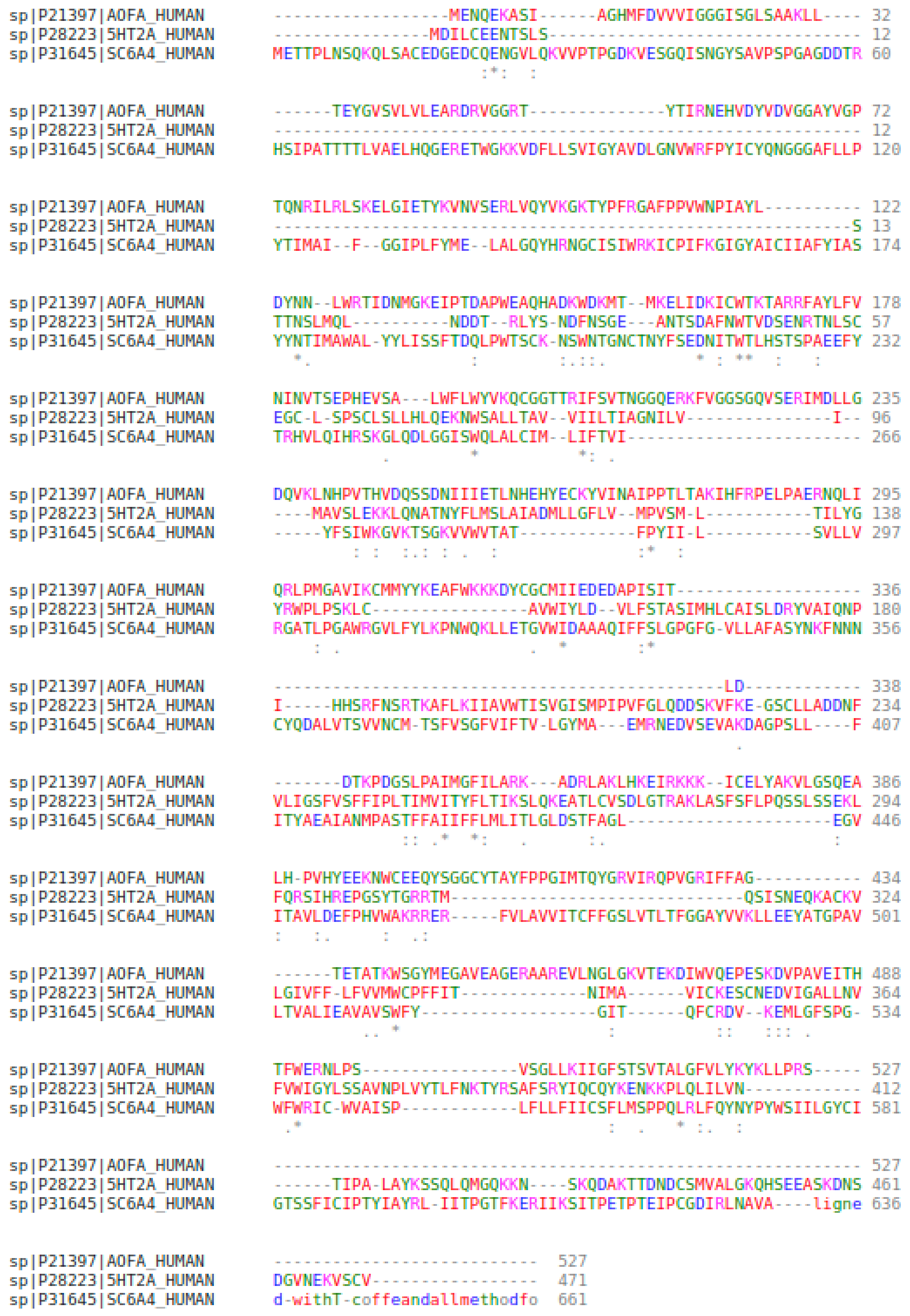

Figure S4: In the figure S4 are shown the results of the multiple sequence alignment among the serotonin target proteins evaluated. Only 15 residues are conserved (*).

Figure S5: Implemented architecture and essential components and services of 3D-PP.

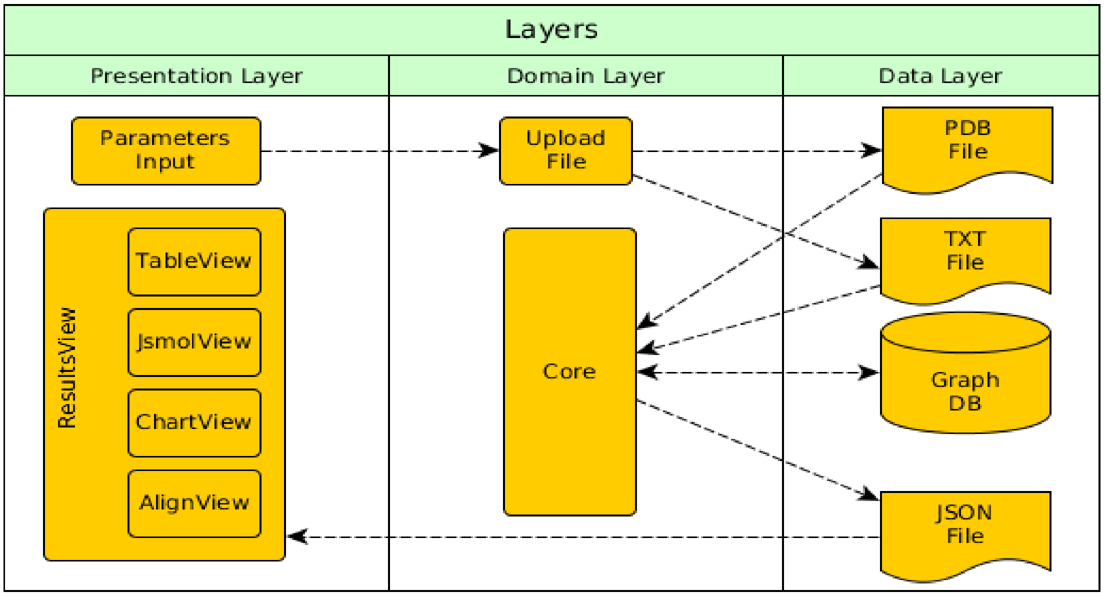

Figure S5: The architecture consists of three main layers: the presentation, domain and data layers, representing the interaction between the essential components of the solution.

This representation is divided into three main layers: the presentation, the domain, and the data layers. I) The presentation layer represents the user's view and the interaction with the domain layer. It consists of two modules: first, ParametersInput which is responsible for obtaining the necessary data to compute the request (Homology model files or PDBids). The second module is the ResultsView, which is composed of the following items: a) a chart showing the frequency of each 3D-pattern discovered (ChartView), b) data tables showing all clusters that belong to each 3D-pattern and all sites that reside into each cluster (TableView), c) a 3D viewer for the structural visualization of the 3D-patterns (JsmolView), d) a picture showing the sequence-based alignment of the sites forming each 3D-pattern (AlignView), e) a graph showing the RMSD of the sites of a cluster. II) The domain layer represents the core of 3D-PP and denotes the communication link between the presentation and data layers. This layer has the following components: a) UploadFile for uploading the file with the PDBids or the homology models delivered by the user, b) Core, for the processing of each protein, the discovering of 3D-patterns and cluster generation, the creation of graph databases, the preparation in JSON format of the results. III) The data layer has three components which are responsible for store the data: a) the PDBFile represents the protein structures under analysis, b) the GraphDB stores all information generated in the Domain Layer, c) the JSONFile contains the processed results of 3D-PP. This component delivers the results to the ResultsView of the Presentation layer.

Figure S6: Graph Database Models.

A

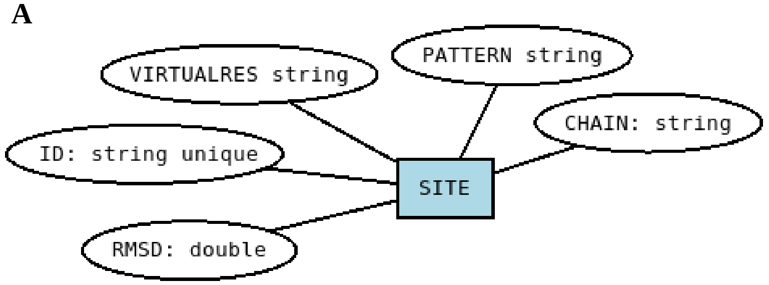

Figure S6A: The model of the graph database implemented for each protein processed.

B

.

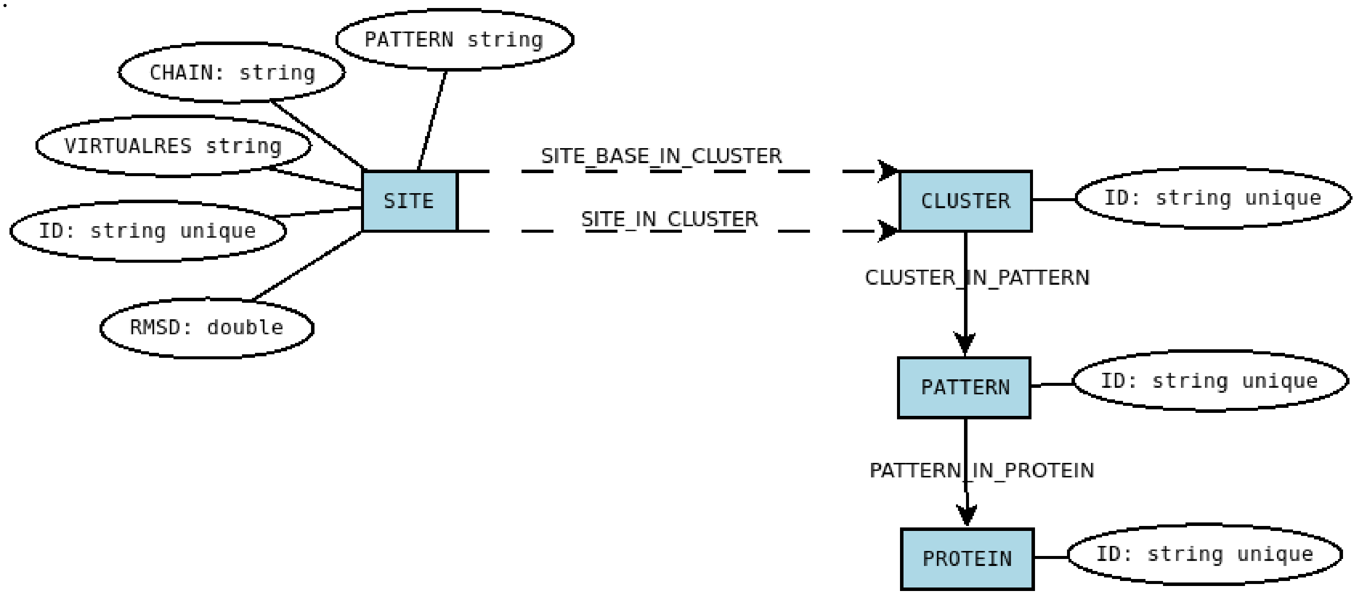

Figure S6B: The graph database model that unifies all the sites found in all proteins.

Table S1: Comparative table showing the occurrence of sites in the original protein structure (pdbSUM), in the primary sequence (C-x(8)-C-x(5)-C-x(3)-H) and in the results obtained with 3D-PP.

PDB Bid	Resolution	Pos.	Sequence C-x(8)-C-x(5)-C-x(3)-H	Fasta from PDB	Site PDB (3D-PP)	pdbSUM	Seq.	3d-pp	Pdb SUM (LigPlot)	Comments
1M9O	N/A	95 – 123	RYKTELCRTYSESGRCRYGAKCQFAHGLG	A:GSHMTTSSRYKTELCRTYSESGRCRYGAKCQFAHGLGELRQANRHPKYKTELCHKFKLQGRCPYGSRCHFIHNPTED	A:CYS15:CYS24:CYS30:HIS34	A:CYS15:CYS24:CYS30:HIS34	Yes	Yes	Yes	
1RGO	N/A	153 – 181	RYKTELCRPFEESGTCKYGEKCQFAHGFH	A:STRYKTELCRPFEESGTCKYGEKCQFAHGFHELRSLTRHPKYKTELCRTFHTIGFCPYGPRCHFIHNADE	A:CYS159:CYS168:CYS174:HIS178	A:CYS159:CYS168:CYS174:HIS178	Yes	Yes	Yes	
		191 – 219	KYKTELCRTFHTIGFCPYGPRCHFIHNAD		A:CYS197:CYS206:CYS212:HIS216	A:CYS197:CYS206:CYS212:HIS216	Yes	Yes	Yes	
2CQE	N/A	419 – 446	PKKRELCKFYITGFCARAENCPYMHGDF	A:GSSGSSGELPKKRELCKFYITGFCARAENCPYMHGDFPCKLYHTTGNCINGDDCMFSHDPLTEETRELLDKMLADDAEAGAEDEKEVEELKKSGPSSG	A:CYS437:CYS445:CYS451:HIS455	A:CYS437:CYS445:CYS451:HIS455	Yes	Yes	Yes	
		447 – 470	PCKLYHTTGNCINGDDCMFSHDPL		A:CYS460:CYS469:CYS475:HIS479	A:CYS460:CYS469:CYS475:HIS479	Yes	Yes	Yes	
2D9M	N/A	906 – 928	ICDRYMNGTCPEGNSCKFAHGNA	A:GSSGSSGQYCWQHRFPTGYFSICDRYMNGTCPEGNSCKFAHGNAELHEWEERRDALKMKLNKASGPSSG	A:CYS907:CYS915:CYS921:HIS925	A:CYS907:CYS915:CYS921:HIS925	Yes	Yes	Yes	
2D9N	N/A	62 – 89	GEKTVVCKHWLRGLCKKGDQCEFLHEYD	A:GSSGSSGEKTVVCKHWLRGLCKKGDQCEFLHEYDMTKMPECYFYSKFGECSNKECPFLHIDPESKIKDCPWSGPSSG	A:CYS68:CYS76:CYS82:HIS70	X	No	Yes	No	New site.
		90 – 117	MTKMPECYFYSKFGECSNKECPFLHIDP		A:CYS68:CYS76:CYS82:HIS86	A:CYS68:CYS76:CYS82:HIS86	Yes	Yes	Yes	
					A:CYS105:CYS110:CYS96:HIS114	A:CYS96:CYS105:CYS110:HIS114	Yes	Yes	Yes	
2E5S	N/A	176 – 204	TDKLEVCREFQRGNCARGETDCRFAHPAD	A:GSSGSSGSTATQKLLRTDKLEVCREFQRGNCARGETDCRFAHPADSTMIDTSDNTVTVCMDYIKGRCMREKCKYFHPPAHLQAKIKAAQHQANQAAVA	A:CYS23:CYS31:CYS38:HIS42	A:CYS23:CYS31:CYS38:HIS42	Yes	Yes	Yes	
		212 – 238	DNTVTVCMDYIKGRCMREKCKYFHPPA		A:CYS59:CYS67:CYS72:HIS76	A:CYS59:CYS67:CYS72:HIS76	Yes	Yes	Yes	
2FC6	N/A	294 – 322	PHPTSICDNFSAYGWCPLGPQCPQSHDID	A:GSSGSSGCCLPPATHRPHPTSICDNFSAYGWCPLGPQCPQSHDISGPSSG	A:CYS23:CYS32:CYS38:HIS42	A:CYS23:CYS32:CYS38:HIS42	Yes	Yes	Yes	

2RHK	1.95 A	62 – 89	GEKTVVCKHWLRGLCKKGDQCEFLHEYD	C:MGHHHHHHSHMSGEKTVVCKHWLRGLCKKGDQCEFLHEYDMTKMSECYFYSKFGECSNKECPFLHIDPESKI<br><br>D:MGHHHHHHSHMSGEKTVVCKHWLRGLCKKGDQCEFLHEYDMTKMSECYFYSKFGECSNKECPFLHIDPESKI	C:CYS105:CYS110:CYS96:HIS114	C:CYS96:CYS105:CYS110:HIS114	Yes	Yes	Yes	
		90 – 117	MTKMPECYFYSKFGECSNKECPFLHIDP		C:CYS68:CYS76:CYS82:HIS86	C:CYS68:CYS76:CYS82:HIS86	Yes	Yes	Yes	
					D:CYS105:CYS110:CYS96:HIS114	D:CYS96:CYS105:CYS110:HIS114	Yes	Yes	Yes	
					D:CYS68:CYS76:CYS82:HIS86	D:CYS68:CYS76:CYS82:HIS86	Yes	Yes	Yes	
2RPP	N/A	13 – 41	WLTLEVCRQFQRGTCSRSDEECKFAHPPK	A:GSSGSSGPVRDTKWLTLEVCRQFQRGTCSRSDEECKFAHPPKSCQVENGRVIACFDSLKGRCSRENCKYLHPPTHLKTQLEINSGPSSG	A:CYS26:CYS34:CYS41:HIS45	A:CYS26:CYS34:CYS41:HIS45	Yes	Yes	Yes	
		47 – 73	NGRVIACFDSLKGRCSRENCKYLHPPT		A:CYS60:CYS68:CYS73:HIS77	A:CYS60:CYS68:CYS73:HIS77	Yes	Yes	Yes	
3D2N	2.7A	13 – 41	WLTLEVCREFQRGTCSRPDTECKFAHPSK	A:SRDTKWLTLEVCREFQRGTCSRPDTECKFAHPSKSCQVENGRVIACFDSLKGRCSRENCKYLHPPPHLKTQLEINGRNNLIQQ	A:CYS19:CYS27:CYS34:HIS38	A:CYS19:CYS27:CYS34:HIS38	Yes	Yes	Yes	
		47 – 73	NGRVIACFDSLKGRCSRENCKYLHPPP		A:CYS53:CYS61:CYS66:HIS70	A:CYS53:CYS61:CYS66:HIS70	Yes	Yes	Yes	
3D2Q	1.5A	179 – 207	TDRLEVCREYQRGNCNRGENDCRFAHPAD	A:SRTDRLEVCREYQRGNCNRGENDCRFAHPADSTMIDTNDNTVTVCMDYIKGRCSREKCKYFHPPAHLQAK<br><br>B:SRTDRLEVCREYQRGNCNRGENDCRFAHPADSTMIDTNDNTVTVCMDYIKGRCSREKCKYFHPPAHLQAK<br><br>C:SRTDRLEVCREYQRGNCNRGENDCRFAHPADSTMIDTNDNTVTVCMDYIKGRCSREKCKYFHPPAHLQAK<br><br>D:SRTDRLEVCREYQRGNCNRGENDCRFAHPADSTMIDTNDNTVTVCMDYIKGRCSREKCKYFHPPAHLQAK	A:CYS185:CYS193:CYS200:HIS204<br>A:CYS221:CYS229:CYS234:HIS238	A:CYS185:CYS193:CYS200:HIS204<br>A:CYS221:CYS229:CYS234:HIS238	Yes	Yes	Yes	
		215 – 241	DNTVTVCMDYIKGRCSREKCKYFHPPA		B:CYS185:CYS193:CYS200:HIS204<br>B:CYS221:CYS229:CYS234:HIS238	B:CYS185:CYS193:CYS200:HIS204<br>B:CYS221:CYS229:CYS234:HIS238	Yes	Yes	Yes	
					C:CYS185:CYS193:CYS200:HIS204<br>C:CYS221:CYS229:CYS234:HIS238	C:CYS185:CYS193:CYS200:HIS204<br>C:CYS221:CYS229:CYS234:HIS238	Yes	Yes	Yes	
					D:CYS185:CYS193:CYS200:HIS204<br>D:CYS221:CYS229:CYS234:HIS238	D:CYS185:CYS193:CYS200:HIS204<br>D:CYS221:CYS229:CYS234:HIS238	Yes	Yes	Yes	
3D2S	1.7A	179 – 207	TDRLEVCREYQRGNCNRGENDCRFAHPAD	A:SRTDRLEVCREYQRGNCNRGENDCRFAHPADSTMIDTNDNTVTVCMDYIKGRCSREKCKYFHPPAHLQAK	A:CYS185:CYS193:CYS200:HIS204<br>A:CYS221:CYS229:CYS234:HIS238	A:CYS185:CYS193:CYS200:HIS204<br>A:CYS221:CYS229:CYS234:HIS238	Yes	Yes	Yes	
		215 –	DNTVTVCMDYIKGRCSREKCKYFHPPA		B:CYS185:CYS193:CYS200:HIS204<br>B:CYS221:CYS229:CYS234:HIS238	B:CYS185:CYS193:CYS200:HIS204<br>B:CYS221:CYS229:CYS234:HIS238	Yes	Yes	Yes	

		241		B:SRTDRLEVCREYQRG<br>NCNRGENDCRFAHPAD<br>STMIDTNDNTVTVCMDY<br>IKGRCSREKCKYFHPPA<br>HLQAK<br><br>C:SRTDRLEVCREYQRG<br>NCNRGENDCRFAHPAD<br>STMIDTNDNTVTVCMDY<br>IKGRCSREKCKYFHPPA<br>HLQAK<br><br>D:SRTDRLEVCREYQRG<br>NCNRGENDCRFAHPAD<br>STMIDTNDNTVTVCMDY<br>IKGRCSREKCKYFHPPA<br>HLQAK	C:CYS185:CYS193:CYS200:HIS204<br>C:CYS221:CYS229:CYS234:HIS238	C:CYS185:CYS193:CYS200:HIS204<br>C:CYS221:CYS229:CYS234:HIS238	Yes	Yes	Yes	
					D:CYS185:CYS193:CYS200:HIS204<br>D:CYS221:CYS229:CYS234:HIS238	D:CYS185:CYS193:CYS200:HIS204<br>D:CYS221:CYS229:CYS234:HIS238	Yes	Yes	Yes	
3<br>J<br>B<br>9	3.6A	111<br>–<br>138	NPGSFFCLYFARGMCSE<br>GSKCEYLHRLP	Y:MSENGLEQEVTVEEK<br>NNDVTEKILVEGEKSKE<br>YEETPRKVKIVKRKKQP<br>ARKQIETRPEYEMEPEQ<br>PGQVYNLWYNKWSGG<br>MRQDPLKSQVKSETRC<br>VISRDSGYTKADKNPGS<br>FFCLYFARGMCSEGSKC<br>EYLHRLPKDTDFFNANV<br>DCFGREKHADYRDDMG<br>GVGSFLRQNYTLYVGGI<br>TPTDDIEEIVSRHFAEW<br>GDIERIRVLNSRGIAFITY<br>LNEANAQFAKEAMAHQ<br>SLDHDECLNVRWATTDP<br>NPASQARNQRRLEERA<br>ANAVKKLLPKQFLLDLEE<br>TKNGKXXXXXXXXXXXX<br>XXXXXXXXXXXXXXXX<br>XXXXXXXXXXXXXXXX<br>XXXXXXXXXXXXXXXX<br>XXXXXXXXXXXXXXXX<br>XXXXXXXXXXXXXXXX<br>XX	e:CYS102:CYS105:CYS137:HIS136	X	No	Yes	No	New site.
					e:CYS105:CYS117:CYS134:HIS136	X	No	Yes	No	New site.
					e:CYS105:CYS134:CYS137:HIS136	X	No	Yes	No	New site.
					Y:CYS117:CYS125:CYS131:HIS135	Y:CYS117:CYS125:CYS131:HIS135	Yes	Yes	Yes	
3<br>T<br>P<br>2	2.4A	67 –<br>94	DGQLFFCLFFAKGMCCL<br>GPKCEYLHHIP	A:GAMTSWRDKSAKVQ<br>VKESELPSSIPAQTGLTF<br>NIWYNKWSQGFAGNTR<br>FVSPFALQPQLHSGKTR<br>GDNDGQLFFCLFFAKG<br>MCCLGPKCEYLHHIPDE<br>EDIGKLALRTEVLDCFG<br>REKFADYREDMGGIGSF<br>RKKNKTLYVGGIDGALN<br>SKHLKPAQIESRIRFVFS	A:CYS73:CYS81:CYS87:HIS91	A:CYS73:CYS81:CYS87:HIS91	Yes	Yes	Yes	
					B:CYS73:CYS81:CYS87:HIS91	B:CYS73:CYS81:CYS87:HIS91	Yes	Yes	Yes	

				RLGDIDRIRYVESKNCG FVKFKYQANAEFAKEAM SNQTLLLPSDKEWDDR REGTGLLVKWAN<br><br>B:GAMTSWRDKSAKVQ VKESELPSSIPAQTGLTF NIWYNKWSQGFAGNTR FVSPFALQPQLHSGKTR GDNDGQLFFCLFFAKG MCCLGPKCEYLHHIPDE EDIGKLALRTEVLDCFG REKFADYREDMGGIGSF RKKNKTLYVGGIDGALN SKHLKPAQIESRIRFVFS RLGDIDRIRYVESKNCG FVKFKYQANAEFAKEAM SNQTLLLPSDKEWDDR REGTGLLVKWAN						
3 U 1 L	1.64 A	67 – 94	DGQLFFCLFFAKGMCCL GPKCEYLHHIP	A:MTSWRDKSAKVQVKE SELPSSIPAQTGLTFNIW YNKWSQGFAGNTRFVS PFALQPQLHSGKTRGD NDGQLFFCLFFAKGMC CLGPKCEYLHHIPDEEDI GKLALRTEVLDCFGREK FADYREDMGGIGSFRKK NKTLYVGGIDGALNSKH LKPAQIESRIRFVFSRLG DIDRIRYVESKNCGFVKF KYQANAEFAKEAMSNQ TLLLPSDKEWDDRREGT GLLVKWANEDPDPAAQ KRLQE	A:CYS73:CYS81:CYS87:HIS91	A:CYS73:CYS81:CYS87:HIS91	Yes	Yes	Yes	
3 U 1 M	1.95 A	67 – 94	DGQLFFCLFFAKGMCCL GPKCEYLHHIP	A:MTSWRDKSAKVQVKE SELPSSIPAQTGLTFNIW YNKWSQGFAGNTRFVS PFALQPQLHSGKTRGD NDGQLFFCLFFAKGMC CLGPKCEYLHHIPDEEDI GKLALRTEVLDCFGREK FADYREDMGGIGSFRKK NKTLYVGGIDGALNSKH LKPAQIESRIRFVFSRLG DIDRIRYVESKNCGFVKF KYQANAEFAKEAMSNQ TLLLPSDKEWDDRREGT GLLVKWANEDPDPAAQ KRLQE	A:CYS73:CYS81:CYS87:HIS91	A:CYS73:CYS81:CYS87:HIS91	Yes	Yes	Yes	
3 U	1.8A	73 – 86	CRRKYCQRPCDSLH	A:GPLGMADPGVCCFIT KILCAHGGRMTLEELLG	A:CYS106:CYS88:CYS96:HIS110	A:CYS106:CYS88:CYS96:HIS110	Yes	Yes	Yes	

9G		88 – 110	CKLNLLGRCHYAQSQRNLCKYSH	EIRLPEAQLYELLETAGPDRFVLLETGGQAGITRSVVATTRARVCRRKYCQRPCDSLHLCKLNLLGRCHYAQSQRNLCKYSHDVLSEQNFQILKNHELSGLNQEELACLLVQSDPFFLPEICKSYKGEGRKQTCGQPQPCERLHICEHFTRGNCSYLNCLRSHNLMDRKVLTIMREHGLSPDVVQNIQDICNNKHAR	A:CYS73:CYS78:CYS82:HIS86	A:CYS73:CYS78:CYS82:HIS86	Yes	Yes	Yes	
		150 – 172	CKSYKGEGRKQTCGQPQPCERLH		A:CYS174:CYS182:CYS187:HIS191	A:CYS174:CYS182:CYS187:HIS191	Yes	Yes	Yes	
		169 – 193	ERLHICEHFTRGNCSYLNCLRSHNL		A:CYS150:CYS162:CYS168:HIS172	A:CYS150:CYS162:CYS168:HIS172	Yes	Yes	Yes	
4C3B	2.95 A	1 – 28	MSRRNPCKFEIRGHCLNGKRCHFSHNYF	A:B:C:D:E:F:G:H:I:J:K:L:M:N:O:P<br><br>GPLGSMSRRNPCKFEIRGHCLNGKRCHFSHNYFEWPPHALLVRQNFMLNRILKSMDKSIDTLSEISGAAELDRTEEYALGVVGVLESYIGSINNITKQSACVAMSKLLTELNSDDIKKLRDNEELNSPKIRVYNTVISYIESNRKNNKQTIHLLKRLPADVLKKTIKNTLDIHKSITINNPKESTVSDTNDHAKNNDTT	A:CYS15:CYS21:CYS7:HIS25	A:CYS15:CYS21:CYS7:HIS25	Yes	Yes	Yes	
					B:CYS15:CYS21:CYS7:HIS25	B:CYS15:CYS21:CYS7:HIS25	Yes	Yes	Yes	
					C:CYS15:CYS21:CYS7:HIS25	C:CYS15:CYS21:CYS7:HIS25	Yes	Yes	Yes	
					D:CYS15:CYS21:CYS7:HIS25	D:CYS15:CYS21:CYS7:HIS25	Yes	Yes	Yes	
					E:CYS15:CYS21:CYS7:HIS25	E:CYS15:CYS21:CYS7:HIS25	Yes	Yes	Yes	
					F:CYS15:CYS21:CYS7:HIS25	F:CYS15:CYS21:CYS7:HIS25	Yes	Yes	Yes	
					G:CYS15:CYS21:CYS7:HIS25	G:CYS15:CYS21:CYS7:HIS25	Yes	Yes	Yes	
					H:CYS15:CYS21:CYS7:HIS25	H:CYS15:CYS21:CYS7:HIS25	Yes	Yes	Yes	
					I:CYS15:CYS21:CYS7:HIS25	I:CYS15:CYS21:CYS7:HIS25	Yes	Yes	Yes	
					J:CYS15:CYS21:CYS7:HIS25	J:CYS15:CYS21:CYS7:HIS25	Yes	Yes	Yes	
					K:CYS15:CYS21:CYS7:HIS25	K:CYS15:CYS21:CYS7:HIS25	Yes	Yes	Yes	
					L:CYS15:CYS21:CYS7:HIS25	L:CYS15:CYS21:CYS7:HIS25	Yes	Yes	Yes	
					M:CYS15:CYS21:CYS7:HIS25	M:CYS15:CYS21:CYS7:HIS25	Yes	Yes	Yes	
					N:CYS15:CYS21:CYS7:HIS25	N:CYS15:CYS21:CYS7:HIS25	Yes	Yes	Yes	
					O:CYS15:CYS21:CYS7:HIS25	O:CYS15:CYS21:CYS7:HIS25	Yes	Yes	Yes	
					P:CYS15:CYS21:CYS7:HIS25	P:CYS15:CYS21:CYS7:HIS25	Yes	Yes	Yes	
4C3D	2.52 A	1 – 28	MSRRNPCKFEIRGHCLNGKRCHFSHNYF	A:GPLGSMSRRNPCKFEIRGHCLNGKRCHFSHNYFEWPPHALLVRQNFMLNRILKSMDKSIDTLSEISGAAELDRTEEYALGVVGVLESYIGSINNITKQSACVAMSKLLTELNSDDIKKLRDNEELNSPKIRVYNTVISYIESNRKNNKQTIHLLKRLPADVLKKTIKNTLDIHKSITINNPKESTVSDTNDHAKNNDTT<br><br>B:GPLGSMSRRNPCKFEIRGHCLNGKRCHFSHNY	A:CYS15:CYS21:CYS7:HIS25	A:CYS15:CYS21:CYS7:HIS25 (not ZN → CD)	Yes	Yes	Yes	CD binding site.
					B:CYS15:CYS21:CYS7:HIS25	B:CYS15:CYS21:CYS7:HIS25 (not ZN → CD)	Yes	Yes	Yes	CD binding site.

				FEWPPHALLVRQNFML NRILKSMDKSIDTLSEIS GAAELDRTEEYALGVVG VLESYIGSINNITKQSAC VAMSKLLTELNSDDIKKL RDNEELNSPKIRVYNTVI SYIESNRKNNKQTIHLLK RLPADVLKKTIKNTLDIH KSITINNPKESTVSDTND HAKNNDTT						
4C3E	2.4A	1 – 28	MSRRNPCKFEIRGHCLN GKRCHFSHNYF	A:B:C:D:E:F:G:H:I:J:K:L: M:NO:P<br><br>GPLGSMSRRNPCKFEIR GHCLNGKRCHFSHNYF EWPPHALLVRQNFMLN RILKSMDKSIDTLDEIDG AAELDRTEEYALGVVGV LESYIGSINNITKQSACV AMSKLLTELNSDDIKKLR DNEELNSPKIRVYNTVIS YIESNRKNNKQTIHLLKR LPADVLKKTIKNTLDIHK SITINNPKESTVSDTNDH AKNNDTT	A:CYS15:CYS21:CYS7:HIS25	A:CYS15:CYS21:CYS7:HIS25	Yes	Yes	Yes	
					B:CYS15:CYS21:CYS7:HIS25	B:CYS15:CYS21:CYS7:HIS25	Yes	Yes	Yes	
					C:CYS15:CYS21:CYS7:HIS25	C:CYS15:CYS21:CYS7:HIS25	Yes	Yes	Yes	
					D:CYS15:CYS21:CYS7:HIS25	D:CYS15:CYS21:CYS7:HIS25	Yes	Yes	Yes	
					E:CYS15:CYS21:CYS7:HIS25	E:CYS15:CYS21:CYS7:HIS25	Yes	Yes	Yes	
					F:CYS15:CYS21:CYS7:HIS25	F:CYS15:CYS21:CYS7:HIS25	Yes	Yes	Yes	
					G:CYS15:CYS21:CYS7:HIS25	G:CYS15:CYS21:CYS7:HIS25	Yes	Yes	Yes	
					H:CYS15:CYS21:CYS7:HIS25	H:CYS15:CYS21:CYS7:HIS25	Yes	Yes	Yes	
					I:CYS15:CYS21:CYS7:HIS25	I:CYS15:CYS21:CYS7:HIS25	Yes	Yes	Yes	
					J:CYS15:CYS21:CYS7:HIS25	J:CYS15:CYS21:CYS7:HIS25	Yes	Yes	Yes	
					K:CYS15:CYS21:CYS7:HIS25	K:CYS15:CYS21:CYS7:HIS25	Yes	Yes	Yes	
					L:CYS15:CYS21:CYS7:HIS25	L:CYS15:CYS21:CYS7:HIS25	Yes	Yes	Yes	
					M:CYS15:CYS21:CYS7:HIS25	M:CYS15:CYS21:CYS7:HIS25	Yes	Yes	Yes	
					N:CYS15:CYS21:CYS7:HIS25	N:CYS15:CYS21:CYS7:HIS25	Yes	Yes	Yes	
					O:CYS15:CYS21:CYS7:HIS25	O:CYS15:CYS21:CYS7:HIS25	Yes	Yes	Yes	
					P:CYS15:CYS21:CYS7:HIS25	P:CYS15:CYS21:CYS7:HIS25	Yes	Yes	Yes	
4CYK	N/A	8 – 37	WAKDIPCRNITIYGYCKK EKEGCPFKHSDN	A:MDKINPDWAKDIPCR NITIYGYCKKEKEGCPFK HSDNTTAT	A:CYS14:CYS23:CYS30:HIS34	A:CYS14:CYS23:CYS30:HIS34	Yes	Yes	Yes	
4II1	2.65 A	175 – 201	KSLKPCPFFLEGKCRFK ENCRFSHGQV	A:B:C:D<br><br>MHHHHHHSSGRENLYF QGEEEGEDEEELSGTK VSAPYYSSWGTLEYHN AMVVGTEEAEDGSAGV RVLYLYPTHKSLKPCPFF	A:CYS180:CYS188:CYS194:HIS198	A:CYS180:CYS188:CYS194:HIS198	Yes	Yes	Yes	
					B:CYS180:CYS188:CYS194:HIS198	B:CYS180:CYS188:CYS194:HIS198	Yes	Yes	Yes	
					C:CYS180:CYS188:CYS194:HIS198	C:CYS180:CYS188:CYS194:HIS198	Yes	Yes	Yes	
					D:CYS180:CYS188:CYS194:HIS198	D:CYS180:CYS188:CYS194:HIS198	Yes	Yes	Yes	

				LEGKCRFKENCRFSHGQVVSLDELRPFQDPDLSSLQAGSACLAKHQDGLWHAARITDVDNGYYTVKFDSLLLREAVVEGDGILPP						
4YH8	1.7A	12 – 40	EQDKVNCSFYYKIGACRHGERCSRKHVKP	A:MASHLASIYGTEQDKVNCSFYYKIGACRHGERCSRKHVKPNFSQTILCPNMYKNPIHEPNGKKFTQRELAEQFDAFYEDMFCEFSKYGEVEQLVVCDNVGDHLVGNVYVRFKYEESAQNAIDDLNSRWYSQRPVYAELSPVTDFREACCRQHETSECQRGGLCNFMHAKKPSPQLLRDLVLAQRKYLALNAAEEMKKEPNSDSTNRWVSVTAERKN	A:CYS149:CYS157:CYS163:HIS167	A:CYS149:CYS157:CYS163:HIS167	Yes	Yes	Yes	
		143 – 170	DFREACCRQHETSECQRGGLCNFMHAKK		A:CYS18:CYS27:CYS33:HIS37	A:CYS18:CYS27:CYS33:HIS37	Yes	Yes	Yes	
5ELH	1.8A	84 – 113	YSPDVYCTKYDEATGLCPEGDECPFLHRTT	A:SPQHYTYLKEFRTEQCPLFVQHKCTQHRPYTCFHWHFVNQRRRRSIRRRDGTFNYSPDVYCTKYDEATGLCPEGDECPFLHRTTGDTERRYHLRYYKTGICIHETDSKGNCTKNGLHCAFAHGPHDLRSPVYDIRELQAMEALQN<br><br>B:SPQHYTYLKEFRTEQCPLFVQHKCTQHRPYTCFHWHFVNQRRRRSIRRRDGTFNYSPDVYCTKYDEATGLCPEGDECPFLHRTTGDTERRYHLRYYKTGICIHETDSKGNCTKNGLHCAFAHGPHDLRSPVYDIRELQAMEALQN	A:CYS130:CYS140:CYS147:HIS151	A:CYS130:CYS140:CYS147:HIS151	Yes	Yes	Yes	
		124 – 154	YYKTGICIHETDSKGNCTKNGLHCAFAHGPH		A:CYS100:CYS106:CYS90:HIS110	A:CYS100:CYS106:CYS90:HIS110	Yes	Yes	Yes	
					A:CYS45:CYS53:CYS61:HIS65	A:CYS45:CYS53:CYS61:HIS65	Yes	Yes	Yes	
					B:CYS130:CYS140:CYS147:HIS132	X	No	Yes	No	New site.
					B:CYS100:CYS106:CYS90:HIS110	B:CYS100:CYS106:CYS90:HIS110	Yes	Yes	Yes	
					B:CYS130:CYS140:CYS147:HIS151	B:CYS130:CYS140:CYS147:HIS151	Yes	Yes	Yes	
					B:CYS45:CYS53:CYS61:HIS65	B:CYS45:CYS53:CYS61:HIS65	Yes	Yes	Yes	
5ELK	2.3A	215 – 241	NYKTEPCKKPPRLCRQGYACPYYHNSK	A:SPRWQETAYVLGNYKTEPCKKPPRLCRQGYACPYYHNSKDRRRSPRKHKYRSSPCPNVKHGDEWGDPGKCENGDACQYCHTRTEQQFHPEIYKSTKCNDMQQAGSCPRGPFCAFAHIEPPPLSDDVQPSSAVS	A:CYS299:CYS308:CYS314:HIS318	A:CYS299:CYS308:CYS314:HIS318	Yes	Yes	Yes	
		251 – 285	KYRSSPCPNVKHGDEWGDPGKCENGDACQYCHTRT		A:CYS257:CYS272:CYS278:HIS282	A:CYS257:CYS272:CYS278:HIS282	Yes	Yes	Yes	
		293 – 321	IYKSTKCNDMQQAGSCPRGPFCAFAHIEP		A:CYS221:CYS228:CYS234:HIS238	A:CYS221:CYS228:CYS234:HIS238	Yes	Yes	Yes	
5G	3.4A	67 – 94	DGQLFFCLFFAKGMCCLGPKCEYLHHIP	R:MTSWRDKSAKVQVKESELPSSIPAQTGLTFNI	R:CYS73:CYS81:CYS87:HIS91	R:CYS73:CYS81:CYS87:HIS91	Yes	Yes	Yes	

M<br>K				WYNKWSQGFAGNTRFV<br>SPFALQPQLHSGKTRGD<br>NDGQLFFCLFFAKGMC<br>CLGPKCEYLHHIPDEEDI<br>GKLALRTEVLDCFGREK<br>FADYREDMGGIGSFRKK<br>NKTLYVGGIDGALNSKH<br>LKPAQIESRIRFVFSRLG<br>DIDRIRYVESKNCGFVKF<br>KYQANAEFAKEAMSNQ<br>TLLLPSDKEWDDRREGT<br>GLLVKWANEDPDPAAQ<br>KRLQEELKLESLNMMVH<br>LINNNTNSAGTEVNNKN<br>NERLDRTFPEASVDNVK<br>KRLLPLDNGMESDDFIE<br>KLKKVKKNISRENISSKP<br>SVGKLGGPLLDYLSSDE<br>D						
					T:CYS108:CYS145:CYS148:HIS147	X	No	Yes	No	New site.
					T:CYS108:CYS120:CYS145:HIS147	X	No	Yes	No	New site.
5<br>L<br>J<br>3	3.8A	67 – 94	DGQLFFCLFFAKGMCCL GPKCEYLHHIP	M:MTSWRDKSAKVQVK<br>ESELPSSIPAQTGLTFNI<br>WYNKWSQGFAGNTRFV<br>SPFALQPQLHSGKTRGD<br>NDGQLFFCLFFAKGMC<br>CLGPKCEYLHHIPDEEDI<br>GKLALRTEALDCFGREK<br>FADYREDMGGIGSFRKK<br>NKTLYVGGIDGALNSKH<br>LKPAQIESRIRFVFSRLG<br>DIDRIRYVESKNCGFVKF<br>KYQANAEFAKEAMSNQ<br>TLLLPSDKEWDDRREGT<br>GLLVKWANEDPDPAAQ<br>KRLQEELKLESLNMMVH<br>LINNNTNSAGTEVNNKN<br>NERLDRTFPEASVDNVK<br>KRLLPLDNGMESDDFIE<br>KLKKVKKNISRENISSKP<br>SVGKLGGPLLDYLSSDE<br>D	L:CYS108:CYS145:CYS148:HIS147	X	No	Yes	No	New site.
					L:CYS108:CYS120:CYS145:HIS147	X	No	Yes	No	New site.
					L:CYS105:CYS108:CYS148:HIS147	X	No	Yes	No	New site.
					M:CYS73:CYS81:CYS87:HIS91	M:CYS73:CYS81:CYS87:HIS91	Yes	Yes	Yes	
5<br>L<br>J<br>5	3.8A	67 – 94	DGQLFFCLFFAKGMCCL GPKCEYLHHIP	M:MTSWRDKSAKVQVK<br>ESELPSSIPAQTGLTFNI<br>WYNKWSQGFAGNTRFV<br>SPFALQPQLHSGKTRGD<br>NDGQLFFCLFFAKGMC<br>CLGPKCEYLHHIPDEEDI<br>GKLALRTEVLDCFGREK<br>FADYREDMGGIGSFRKK<br>NKTLYVGGIDGALNSKH<br>LKPAQIESRIRFVFSRLG<br>DIDRIRYVESKNCGFVKF<br>KYQANAEFAKEAMSNQ	L:CYS108:CYS145:CYS148:HIS147	X	No	Yes	No	New site.
					L:CYS108:CYS120:CYS145:HIS147	X	No	Yes	No	New site.
					L:CYS105:CYS108:CYS148:HIS147	X	No	Yes	No	New site.
					M:CYS73:CYS81:CYS87:HIS91	M:CYS73:CYS81:CYS87:HIS91	Yes	Yes	Yes	

				TLLLPSDKEWDDRREGT<br>GLLVKWANEDPDPAAQ<br>KRLQEELKLESLNMMVH<br>LINNNTNSAGTEVNNKN<br>NERLDRTFPEASVDNVK<br>KRLLPLDNGMESDDFIE<br>KLKKVKKNISRENISSKP<br>SVGKLGGPLLDYLSSDE<br>D						
5<br>L<br>Q<br>W	5.8A	67 –<br>94	DGQLFFCLFFAKGMCCL<br>GPKCEYLHHIP	F:MTSWRDKSAKVQVKE<br>SELPSSIPAQTGLTFNIW<br>YNKWSQGFAGNTRFVS<br>PFALQPQLHSGKTRGD<br>NDGQLFFCLFFAKGMC<br>CLGPKCEYLHHIPDEEDI<br>GKLALRTEVLDCFGREK<br>FADYREDMGGIGSFRKK<br>NKTLYVGGIDGALNSKH<br>LKPAQIESRIRFVFSRLG<br>DIDRIRYVESKNCGFVKF<br>KYQANAEFAKEAMSNQ<br>TLLLPSDKEWDDRREGT<br>GLLVKWANEDPDPAAQ<br>KRLQEELKLESLNMMVH<br>LINNNTNSAGTEVNNKN<br>NERLDRTFPEASVDNVK<br>KRLLPLDNGMESDDFIE<br>KLKKVKKNISRENISSKP<br>SVGKLGGPLLDYLSSDE<br>D						
5<br>M<br>P<br>S	3.8A	67 –<br>94	DGQLFFCLFFAKGMCCL<br>GPKCEYLHHIP	M:MTSWRDKSAKVQVK<br>ESELPSSIPAQTGLTFNI<br>WYNKWSQGFAGNTRFV<br>SPFALQPQLHSGKTRGD<br>NDGQLFFCLFFAKGMC<br>CLGPKCEYLHHIPDEEDI<br>GKLALRTEVLDCFGREK<br>FADYREDMGGIGSFRKK<br>NKTLYVGGIDGALNSKH<br>LKPAQIESRIRFVFSRLG<br>DIDRIRYVESKNCGFVKF<br>KYQANAEFAKEAMSNQ<br>TLLLPSDKEWDDRREGT<br>GLLVKWANEDPDPAAQ<br>KRLQEELKLESLNMMVH<br>LINNNTNSAGTEVNNKN<br>NERLDRTFPEASVDNVK<br>KRLLPLDNGMESDDFIE<br>KLKKVKKNISRENISSKP<br>SVGKLGGPLLDYLSSDE<br>D	L:CYS108:CYS145:CYS148:HIS147	X	No	Yes	No	New site.
					M:CYS73:CYS81:CYS87:HIS91	M:CYS73:CYS81:CYS87:HIS91	Yes	Yes	Yes	

5MQ0	4.17 A	67 – 94	DGQLFFCLFFAKGMCCLGPKCEYLHHIP	M:MTSWRDKSAKVQVKESELPSSIPAQTGLTFNIWYNKWSQGFAGNTRFVSPFALQPQLHSGKTRGDNDGQLFFCLFFAKGMCCLGPKCEYLHHIPDEEDIGKLALRTEVLDCFGREKFADYREDMGGIGSFRKKNKTLYVGGIDGALNSKHLKPAQIESRIRFVFSRLGDIDRIRYVESKNCGFVKFKYQANAEFAKEAMSNQTLLLPSDKEWDDRREGTGLLVKWANEDPDPAAQKRLQEELKLESLNMMVHLINNNTNSAGTEVNNKNNERLDRTFPEASVDNVKKRLLPLDNGMESDDFIEKLKKVKKNISRENISSKPSVGKLGGPLLDYLSSDED	L:CYS108:CYS145:CYS148:HIS147	X	No	Yes	No	New site.
					M:CYS73:CYS81:CYS87:HIS91	M:CYS73:CYS81:CYS87:HIS91	Yes	Yes	Yes	
5MQF	5.9A	159 – 186	RNRPHICSFWVKGECKRGEECPYRHEKP	P:MATSLGSNTYNRQNWEDADFPILCQTCLGENPYIRMTKEKYGKECKICARPFTVFRWCPGVRMRFKKTEVCQTCSKLKNVCQTCLLDLEYGLPIQVRDAGLSFKDDMPKSDVNKEYYTQNMEREISNSDGTRPVGMLGKATSTSDMLLKLARTTPYYKRNRPHICSFWVKGECKRGEECPYRHEKPTDPDDPLADQNIKDRYYGINDPVADKLLKRASTMPRLDPPEDKTITTLYVGGLGDTITETDLRNHFYQFGEIRTITVVQRQQCAFIQFATRQAAEVAAEKSFNKLIVNGRRLNVKWGRSQAARGKEKEKDGTTDSGIKLEPVPGLPGALPPPPAAEEEASANYFNLPPSGPPAVVNIALPPPPGIAPPPPPGFGPHMFHPMGPPPPFMRAPGPIHYPSQDPQRMGAHAGKHSSP						
5U6H	N/A	13 – 41	WLTLEVCREFQRGTCSRPDTECKFAHPSK	A:MAVSVTPIRDTKWLTLEVCREFQRGTCSRPDTECKFAHPSKSCQVENGRVIACFDSLKGRCSRENCKYLHPPPHLKTQLEIN	A:CYS19:CYS27:CYS34:HIS38	A:CYS19:CYS27:CYS34:HIS38	Yes	Yes	Yes	
		47 – 73	NGRVIACFDSLKGRCSRENCKYLHPPP			A:CYS53:CYS61:CYS66:HIS70	Yes	No	Yes	

				GRNNLIQQKN						
5U6L	N/A	179 – 207	TDRLEVCREYQRGNCNRGENDCRFAHPAD	A:AQKLMRTDRLEVCREYQRGNCNRGENDCRFAHPADSTMIDTNDNTVTVCMDYIKGRCSREKCKYFHPPAHLQAKIKAAQYQVN	A:CYS221:CYS229:CYS234:HIS238	A:CYS221:CYS229:CYS234:HIS238	Yes	Yes	Yes	
		215 – 241	DNTVTVCMDYIKGRCSREKCKYFHPPA		A:CYS185:CYS193:CYS200:HIS204	A:CYS185:CYS193:CYS200:HIS204	Yes	Yes	Yes	
5U9B	N/A	13 – 41	WLTLEVCREFQRGTCSRPDTECKFAHPSK	A:MAVSVTPIRDTKWLTLEVCREFQRGTCSRPDTECKFAHPSKSCQVENGRVIACFDSLKGRCSRENCKYLHPPPHLKTQLEINGRNNLIQQKN	A:CYS53:CYS61:CYS66:HIS70	A:CYS53:CYS61:CYS66:HIS70	Yes	Yes	Yes	
		47 – 73	NGRVIACFDSLKGRCSRENCKYLHPPP		A:CYS19:CYS27:CYS34:HIS38	A:CYS19:CYS27:CYS34:HIS38	Yes	Yes	Yes	
5WSG	4.0A	67 – 94	DGQLFFCLFFAKGMCCLGPKCEYLHHIP	R:MTSWRDKSAKVQVKESELPSSIPAQTGLTFNIWYNKWSQGFAGNTRFVSPFALQPQLHSGKTRGDNDGQLFFCLFFAKGMCCLGPKCEYLHHIPDEEDIGKLALRTEVLDCFGREKFADYREDMGGIGSFRKKNKTLYVGGIDGALNSKHLKPAQIESRIRFVFSRLGDIDRIRYVESKNCGFVKFKYQANAEFAKEAMSNQTLLLPSDKEWDDRREGTGLLVKWANEDPDPAAQKRLQEELKLESLNMMVHLINNNTNSAGTEVNNKNNERLDRTFPEASVDNVKKRLLPLDNGMESDDFIEKLKKVKKNISRENISSKPSVGKLGGPLLDYLSSDED	R:CYS73:CYS81:CYS87:HIS91	R:CYS73:CYS81:CYS87:HIS91	Yes	Yes	Yes	
					T:CYS108:CYS145:CYS148:HIS147	X	No	Yes	No	New site.
					T:CYS108:CYS120:CYS145:HIS147	X	No	Yes	No	New site.
5Y88	3.7A	67 – 94	DGQLFFCLFFAKGMCCLGPKCEYLHHIP	N:MTSWRDKSAKVQVKESELPSSIPAQTGLTFNIWYNKWSQGFAGNTRFVSPFALQPQLHSGKTRGDNDGQLFFCLFFAKGMCCLGPKCEYLHHIPDEEDIGKLALRTEVLDCFGREKFADYREDMGGIGSFRKKNKTLYVGGIDGALNSKHLKPAQIESRIRFVFSRLGDIDRIRYVESKNCGFVKFKYQANAEFAKEAMSNQTLLLPSDKEWDDRREGTGLLVKWANEDPDPAAQKRLQEELKLESLNMMVHLINNNTNSAGTEVNNKN	L:CYS108:CYS145:CYS148:HIS147	X	No	Yes	No	New site.
					L:CYS108:CYS120:CYS145:HIS147	X	No	Yes	No	New site.
					N:CYS73:CYS81:CYS87:HIS91	N:CYS73:CYS81:CYS87:HIS91	Yes	Yes	Yes	

				NERLDRTFPEASVDNVKKRLLPLDNGMESDDFIEKLKKVKKNISRENISSKPSVGKLGGPLLDYLSSDED						
5YLZ	3.6A	67 – 94	DGQLFFCLFFAKGMCCLGPKCEYLHHIP	N:MTSWRDKSAKVQVKESELPSSIPAQTGLTFNIWYNKWSQGFAGNTRFVSPFALQPQLHSGKTRGDNDGQLFFCLFFAKGMCCLGPKCEYLHHIPDEEDIGKLALRTEVLDCFGREKFADYREDMGGIGSFRKKNKTLYVGGIDGALNSKHLKPAQIESRIRFVFSRLGDIDRIRYVESKNCGFVKFKYQANAEFAKEAMSNQTLLLPSDKEWDDRREGTGLLVKWANEDPDPAAQKRLQEELKLESLNMMVHLINNNTNSAGTEVNNKNNERLDRTFPEASVDNVKKRLLPLDNGMESDDFIEKLKKVKKNISRENISSKPSVGKLGGPLLDYLSSDED	L:CYS108:CYS145:CYS148:HIS147	X	No	Yes	No	New site.
					L:CYS108:CYS120:CYS145:HIS147	X	No	Yes	No	New site.
					N:CYS73:CYS81:CYS87:HIS91	N:CYS73:CYS81:CYS87:HIS91	Yes	Yes	Yes	
5Z58	4.9A	196 – 224	DYQPDICKDYKETGFCGFGDSCKFLHDRS	M:MAEQLSPGKAVDQVCTFLFKKPGRKGAAGRRKRPACDPEPGESGSSSDEGCTVVRPEKKRVTHNPMIQKTRDSGKQKAAYGDLSSEEEEENEPESLGVVYKSTRSAKPVGPEDMGATAVYELDTEKERDAQAIFERSQKIQEELRGKEDDKIYRGINNYQKYMKPKDTSMGNASSGMVRKGPIRAPEHLRATVRWDYQPDICKDYKETGFCGFGDSCKFLHDRSDYKHGWQIERELDEGRYGVYEDENYEVGSDDEEIPFKCFICRQSFQNPVVTKCRHYFCESCALQHFRTTPRCYVCDQQTNGVFNPAKELIAKLEKHRATGEGGASDLPEDPDEDAIPIT	M:CYS202:CYS211:CYS217:HIS221	M:CYS202:CYS211:CYS217:HIS221	Yes	Yes	Yes	
6BK	3.3A	67 – 94	DGQLFFCLFFAKGMCCLGPKCEYLHHIP	G:MTSWRDKSAKVQVKESELPSSIPAQTGLTFNIWYNKWSQGFAGNTRFV	G:CYS73:CYS81:CYS87:HIS91	G:CYS73:CYS81:CYS87:HIS91	Yes	Yes	Yes	
					I:CYS108:CYS145:CYS148:HIS147	X	No	Yes	No	New Site

8				SPFALQPQLHSGKTRGDNDGQLFFCLFFAKGMCCLGPKCEYLHHIPDEEDIGKLALRTEVLDCFGREKFADYREDMGGIGSFRKKNKTLYVGGIDGALNSKHLKPAQIESRIRFVFSRLGDIDRIRYVESKNCGFVKFKYQANAEFAKEAMSNQTLLLPSDKEWDDRREGTGLLVKWANEDPDPAAQKRLQEELKLESLNMMVHLINNNTNSAGTEVNNKNNERLDRTFPEASVDNVKKRLLPLDNGMESDDFIEKLKKVKKNISRENISSKPSVGKLGGPLLDYLSSDED<br><br>O:MNNNSRNNENRSTINRNKRQLQQAKEKNENIHIPRYIRNQPWYYKDTPKEQEGKKPGNDDTSTAEGGEKSDYLVHHRQKAKGGALDIDNNSEPKIGMGIKDEFKLIRPQKMSVRDSHSLSFCRNCGEAGHKEKDCMEKPRKMQKLVPDLNSQKNNGTVLVRATDDDWDSRKDRWYGYSGKEYNELISKWERDKRNKIKGKDKSQTDETLWDTDEEIELMKLELYKDSVGSLKKDDADNSQLYRTSTRLREDKAAYLNDINSTESNYDPKSRLYKTETLGAVDEKSKMFRRHLTGEGLKLNELNQFARSHAKEMGIRDEIEDKEKVQHVLVANPTKYEYLKKKREQEETKQPKIVSIGDLEARKVDGTKQSEEQRNHLKDLYG						
		120 - 137	SFCRNCGEAGHKEKDCME		I:CYS108:CYS120:CYS145:HIS147	X	No	Yes	No	New Site
					I:CYS105:CYS108:CYS148:HIS147	X	No	Yes	No	New Site
					O:CYS122:CYS125:CYS135:HIS130	O:CYS122:CYS125:CYS135:HIS130	Yes	Yes	Yes	
6DNH	3.4A	35 – 61	KSGAAVCEFFLKAACGKGGMCPFRHIS	C:GMQEIIASVDHIKFDLEIAVEQQLGAQPLPFPGMDKSGAAVCEFFLKAACGKGGMCPFRHISGEKTVVCKHWLRGLCKKGDQCEFLHEYDMTKMPECYFYSKFGECSNKECPFLHIDPESKIKDCPWYDRGFCKHGPLCRHRHTRRVICVNYLVGFCPEGPSCKFMH	C:CYS105:CYS110:CYS96:HIS114	X (2C1H)	Yes	Yes	No	New site.
		62 – 89	GEKTVVCKHWLRGLCKKGDQCEFLHEYD		X	C:CYS68:CYS76:CYS82:HIS86	Yes	No	Yes	
		90 – 117	MTKMPECYFYSKFGECSNKECPFLHIDP		C:CYS41:CYS49:CYS55:HIS:59	C:CYS41:CYS49:CYS55:HIS:59	Yes	Yes	Yes	

				PRFELPMGTTEQPPLPQ QTQPPAKQRTPQVIGVM QSQNSSAGNRGPRPLE QVTCYKCGEKGHYANR CTKGHLAFLSGQ						
6EOJ	3.55 A	28 – 59	DPDRPICEFYNSREGPK SCPRGPLCPKKHVLP	B:PSLIHPDTAKYPFKFE PFLRQEYSFSLDPDRPI CEFYNSREGPKSCPRG PLCPKKHVLPIFQNKIVC RHWLRGLCKKNDQCEY LHEYNLRKMPECVFFSK NGYCTQSPDCQYLHIDP ASKIPKCENYEMGFCPL GSSCPRRHIKKVFCQRY MTGFCPLGKDECDMEH PQFIIPDEGSKLRIKRDD EINTRKMDEEKERRLNAI INGEV	B:CYS67:CYS75:CYS81:HIS85	B:CYS67:CYS75:CYS81:HIS85	Yes	Yes	Yes	
		61 – 88	FQNKIVCRHWLRGLCKK NDQCEYLHEYN		B:CYS34:CYS46:CYS52:HIS56	B:CYS34:CYS46:CYS52:HIS56	Yes	Yes	Yes	
6EXN	3.7A	67 – 94	DGQLFFCLFFAKGMCCL GPKCEYLHHIP	M:MTSWRDKSAKVQVK ESELPSSIPAQTGLTFNI WYNKWSQGFAGNTRFV SPFALQPQLHSGKTRGD NDGQLFFCLFFAKGMC CLGPKCEYLHHIPDEEDI GKLALRTEVLDCFGREK FADYREDMGGIGSFRKK NKTLYVGGIDGALNSKH LKPAQIESRIRFVFSRLG DIDRIRYVESKNCGFVKF KYQANAEFAKEAMSNQ TLLLPSDKEWDDRREGT GLLVKWANEDPDPAAQ KRLQEELKLESLNMMVH LINNNTNSAGTEVNNKN NERLDRTFPEASVDNVK KRLLPLDNGMESDDFIE KLKKVKKNISRENISSKP SVGKLGGPLLDYLSSDE D<br><br>c:MNNNSRNNENRSTIN RNKRQLQQAKEKNENIH IPRYIRNQPWYYKDTPK EQEGKKPGNDDTSTAE GGEKSDYLVHHRQKAK GGALDIDNNSEPKIGMGI KDEFKLIRPQKMSVRDS HSLSFCRNCGEAGHKE KDCMEKPRKMQKLVPD LNSQKNNGTVLVRATDD DWDSRKDRWYGYSGK EYNELISKWERDKRNKI	c:CYS122:CYS125:CYS135:HIS130	c:CYS122:CYS125:CYS135:HIS130	Yes	Yes	Yes	
					L:CYS108:CYS145:CYS148:HIS147	X	No	Yes	No	New site.
		120-137	SFCRNCGEAGHKEKDC ME		L:CYS108:CYS120:CYS145:HIS147	X	No	Yes	No	New site.
					L:CYS105:CYS108:CYS148:HIS147	X	No	Yes	No	New site.
					M:CYS73:CYS81:CYS87:HIS91	M:CYS73:CYS81:CYS87:HIS91	Yes	Yes	Yes	

				KGKDKSQTDETLWDTD EEIELMKLELYKDSVGSL KKDDADNSQLYRTSTRL REDKAAYLNDINSTESN YDPKSRLYKTETLGAVD EKSKMFRRHLTGEGLKL NELNQFARSHAKEMGIR DEIEDKEKVQHVLVANP TKYEYLKKKREQEETKQ PKIVSIGDLEARKVDGTK QSEEQRNHLKDLYG						
6FBS	3.07 A	35 – 61	KSGAAVCEFFLKAACGK GGMCPFRHIS	C:MQEIIASVDHIKFDLEI AVEQQLGAQPLPFPGM DKSGAAVCEFFLKAACG KGGMCPFRHISGEKTVV CKHWLRGLCKKGDQCE FLHEYDMTKMPECYFYS KFGECSNKECPFLHIDP ESKIKDCPWYDRGFCK HGPLCRHRHTRRVICVN YLVGFCPEGPSCKFMH PRFELPMGTTEQ	C:CYS105:CYS110:CYS96:HIS114	C:CYS105:CYS110:CYS96:HIS114	Yes	Yes	Yes	
		62 – 89	GEKTVVCKHWLRGLCKK GDQCEFLHEYD		C:CYS68:CYS76:CYS82:HIS86	C:CYS68:CYS76:CYS82:HIS86	Yes	Yes	Yes	
		90 – 117	MTKMPECYFYSKFGECS NKECPFLHIDP		C:CYS41:CYS49:CYS55:HIS59	C:CYS41:CYS49:CYS55:HIS59	Yes	Yes	Yes	
6FF4	3.4A	196 – 224	DYQPDICKDYKETGFCG FGDSCKFLHDRS	t:MAEQLSPGKAVDQVC TFLFKKPGRKGAAGRRK RPACDPEPGESGSSSD EGCTVVRPEKKRVTHN PMIQKTRDSGKQKAAYG DLSSEEEEENEPESLGV VYKSTRSAKPVGPEDM GATAVYELDTEKERDAQ AIFERSQKIQEELRGKED DKIYRGINNYQKYMKPK DTSMGNASSGMVRKGP IRAPEHLRATVRWDYQP DICKDYKETGFCGFGDS CKFLHDRSDYKHGWQI ERELDEGRYGVYEDEN YEVGSDDEEIPFKCFICR QSFQNPVVTKCRHYFC ESCALQHFRTTPRCYVC DQQTNGVFNPAKELIAK LEKHRATGEGGASDLPE DPDEDAIPIT	Q:CYS105:CYS134:CYS137:HIS136	X	No	Yes	No	New site.
					Q:CYS105:CYS117:CYS134:HIS136	X	No	Yes	No	New site.
					t:CYS202:CYS211:CYS217:HIS221	t:CYS202:CYS211:CYS217:HIS221	Yes	Yes	Yes	
6FUW	3.07 A	35 – 61	KSGAAVCEFFLKAACGK GGMCPFRHIS	C:MQEIIASVDHIKFDLEI AVEQQLGAQPLPFPGM DKSGAAVCEFFLKAACG KGGMCPFRHISGEKTVV CKHWLRGLCKKGDQCE FLHEYDMTKMPECYFYS KFGECSNKECPFLHIDP ESKIKDCPWYDRGFCK	C:CYS105:CYS110:CYS96:HIS114	C:CYS105:CYS110:CYS96:HIS114	Yes	Yes	Yes	
		62 – 89	GEKTVVCKHWLRGLCK KGDQCEFLHEYD		C:CYS68:CYS76:CYS82:HIS86	C:CYS68:CYS76:CYS82:HIS86	Yes	Yes	Yes	
		90 – 117	MTKMPECYFYSKFGEC SNKECPFLHIDP		C:CYS41:CYS49:CYS55:HIS59	C:CYS41:CYS49:CYS55:HIS59	Yes	Yes	Yes	

				HGPLCRHRHTRRVICVN YLVGFCPEGPSCKFMH PRFELPMGTTEQ						

Table S2: Matrix of amino acids sequence identity among the Serotonin Transporter (SERT), the Monoamine Oxidase A (MAO-A) and the Serotonin receptor 2A type (5HT2A).

	MAO-A	5HT2A	SERT
**MAO-A**	100%	15%	18%
**5HT2A**	15%	100%	22%
**SERT**	18%	22%	100%

Table S2: In the table are shown the amino acids sequence identity between MAO-A vs SERT, MAO-A vs 5HT2A, and SERT vs 5HT2A.
